# Resident Macrophage‐Orchestrated Immune and Fibroblast Interactions in Immune Checkpoint Inhibitor‐Associated Nephrotoxicity

**DOI:** 10.1002/advs.202505445

**Published:** 2025-08-14

**Authors:** Yanhong Ma, Yang Chen, Qinfan Yao, Yuxi Wang, Nan Ouyang, Fei Han, Guzong Wang, Meifang Wang, Yuan Yuan, Jianghua Chen, Jianzhen Shan, Dajin Chen

**Affiliations:** ^1^ Department of Medical Oncology The First Affiliated Hospital Zhejiang University School of Medicine Hangzhou Zhejiang 310003 China; ^2^ Kidney Disease Center The First Affiliated Hospital Zhejiang University School of Medicine Hangzhou Zhejiang 310003 China; ^3^ Urology and Nephrology Center Department of Nephrology Zhejiang Provincial People's Hospital Affiliated People's Hospital Hangzhou Medical College Hangzhou Zhejiang 310014 China; ^4^ Department of Nephrology The First Affiliated Hospital of Chongqing Medical University Chongqing 400016 China; ^5^ Department of Thoracic Surgery The First Affiliated Hospital Zhejiang University School of Medicine Liangzhu Branch The First People's Hospital of Yuhang District Hangzhou 311100 China; ^6^ Department of Nephrology Hangzhou TCM Hospital Affiliated to Zhejiang Chinese Medical University Hangzhou 310007 China

**Keywords:** CXCL9, immune checkpoint inhibitors, MMP12, nephrotoxicity, resident macrophages

## Abstract

Immune checkpoint inhibitors (ICIs) transform cancer therapy but are often associated with immune‐related adverse events, including ICI‐associated nephrotoxicity (ICI‐AN). The mechanisms driving ICI‐AN remain poorly understood, limiting diagnostic and therapeutic progress. This study integrates imaging mass cytometry, transcriptomics, and murine models to investigate the cellular and molecular mechanisms driving ICI‐AN. Kidney biopsies from ICI‐AN patients show increased resident macrophages, fibroblasts, and CD8⁺ T cells, with resident macrophages exhibiting elevated pro‐inflammatory and pro‐fibrotic markers. In mice, anti‐PD‐1 treatment induces renal injury characterized by immune cell infiltration, tubular injury, and fibrosis. Depletion of resident macrophages reduces CXCL9 and MMP12 expression, alleviating renal injury without compromising anti‐tumor effects. Transcriptomic analysis confirms that inflammatory and fibrotic pathways involving CXCL9 and MMP12 are central to ICI‐AN pathogenesis. Urinary CXCL9 levels are significantly elevated in ICI‐AN patients, correlating with tubular injury and resident macrophage abundance, while MMP12 expression is predominantly localized to resident macrophages. Pharmacological inhibition of MMP12 mitigates renal injury. These results highlight resident macrophages as central drivers of ICI‐AN and identify CXCL9 as a potential diagnostic biomarker and MMP12 as a therapeutic target. This study provides new insights into ICI‐AN pathogenesis and offers strategies to mitigate nephrotoxicity.

## Graphical abstract

Schematic illustration of resident macrophages in Immune checkpoint inhibitor‐associated nephrotoxicity (ICI‐AN). Using an integrated approach (including imaging mass cytometry, transcriptomics, and murine models), this study reveals that resident macrophages drive immune‐stromal interactions in ICI‐AN, promoting fibroblast activation through CXCL9 and MMP12. These findings highlight macrophages as key drivers of ICI‐AN and suggest potential diagnostic and therapeutic targets.

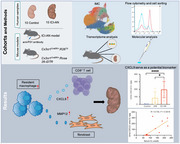

## 1 Introduction

Immune checkpoint inhibitors (ICIs), which target the PD‐1/PD‐L1 and CTLA‐4 pathways, have transformed cancer therapy by enhancing T cell‐mediated immunity. Currently, ≈50% of all cancer patients are eligible for treatment with ICIs.^[^ ^1^ ^]^ These agents have markedly improved survival outcomes across various cancers, being employed in neoadjuvant, adjuvant, and maintenance treatment regimens.^[^ ^2^, ^3^, ^4^ ^]^

However, the remarkable efficacy of ICIs is often accompanied by immune‐related adverse events (irAEs), affecting multiple organs, including the kidneys.^[^ ^5^, ^6^, ^7^ ^]^ These irAEs are often unpredictable, diverse in clinical presentation, and can sometimes be irreversible or pose a threat to life.^[^ ^8^ ^]^ Renal toxicity directly attributed to the ICIs, referred to as immune checkpoint inhibitor‐associated nephrotoxicity (ICI‐AN), occurs in 3–5% of patients. It commonly manifests as acute tubulointerstitial nephritis (TIN), glomerulonephritis, or tubular injury, leading to significant renal dysfunction.^[^ ^7^, ^9^, ^10^ ^]^ ICI‐AN carries significant consequences, potentially resulting in the discontinuation of life‐saving cancer therapy and extended use of immunosuppressive treatments.

Evaluating suspected ICI‐AN requires a comprehensive clinical evaluation including medical history, physical examination, urinalysis, imaging of the urinary tract, and kidney biopsy in severe cases. However, commonly used indicators such as serum creatinine (Cr) and urinary albumin are nonspecific, and there is a lack of reliable biomarkers that reflect the underlying immune mechanisms. This diagnostic uncertainty underscores the need to uncover the molecular and cellular mechanisms driving ICI‐AN.

Current hypotheses regarding the pathogenesis of ICI‐AN are largely derived from preclinical studies. One possible mechanism involves the loss of tolerance to self‐antigens.^[^ ^11^, ^12^ ^]^ Additionally, ICIs can reactivate dormant drug‐specific effector T cells associated with medications such as NSAIDs, proton pump inhibitors (PPIs), and antibiotics. These drugs may act as haptens, triggering immune responses that result in tubular injury.^[^ ^7^, ^13^, ^14^ ^]^ Furthermore, ICIs may induce the production of autoantibodies targeting renal epithelial cells, podocytes, or mesangial cells, a process driven by elevated cytokine levels that create a pro‐inflammatory environment.^[^ ^15^, ^16^ ^]^ Previous studies have highlighted the role of immunological mechanisms, including T cells and macrophages, in driving kidney injury during ICI therapy.^[^ ^17^, ^18^ ^]^ However, the spatial organization, molecular mediators, and functional contributions of these immune cell populations remain poorly understood.

In this study, we used an integrative approach combining imaging mass cytometry (IMC), transcriptomics, and murine models to define the immune architecture and molecular drivers of ICI‐AN. We recognized resident macrophages served as key orchestrators of immune activation and fibrosis, with CXCL9 and MMP12 mediating macrophage‐driven CD8⁺ T cell recruitment and tissue remodeling.

Notably, urinary CXCL9 levels correlated with macrophage abundance and tubular injury, indicating its potential as a non‐invasive, mechanism‐informed diagnostic biomarker. In parallel, MMP12, which was enriched in fibrotic regions, emerged as a key effector of tissue remodeling. Pharmacologic inhibition of MMP12 significantly attenuated tubular damage, and interstitial fibrosis, further supporting its role as a therapeutic target in ICI‐AN.

By linking the clinical observations with mechanistic insights, our findings provide a framework for developing more specific diagnostic tools and targeted strategies to mitigate irAEs without compromising the anti‐tumor efficacy of ICIs.

## 2 Results

### 2.1 Multiplex Imaging Analysis and Histological Evaluation of Kidney Tissues

**Figure 1:**
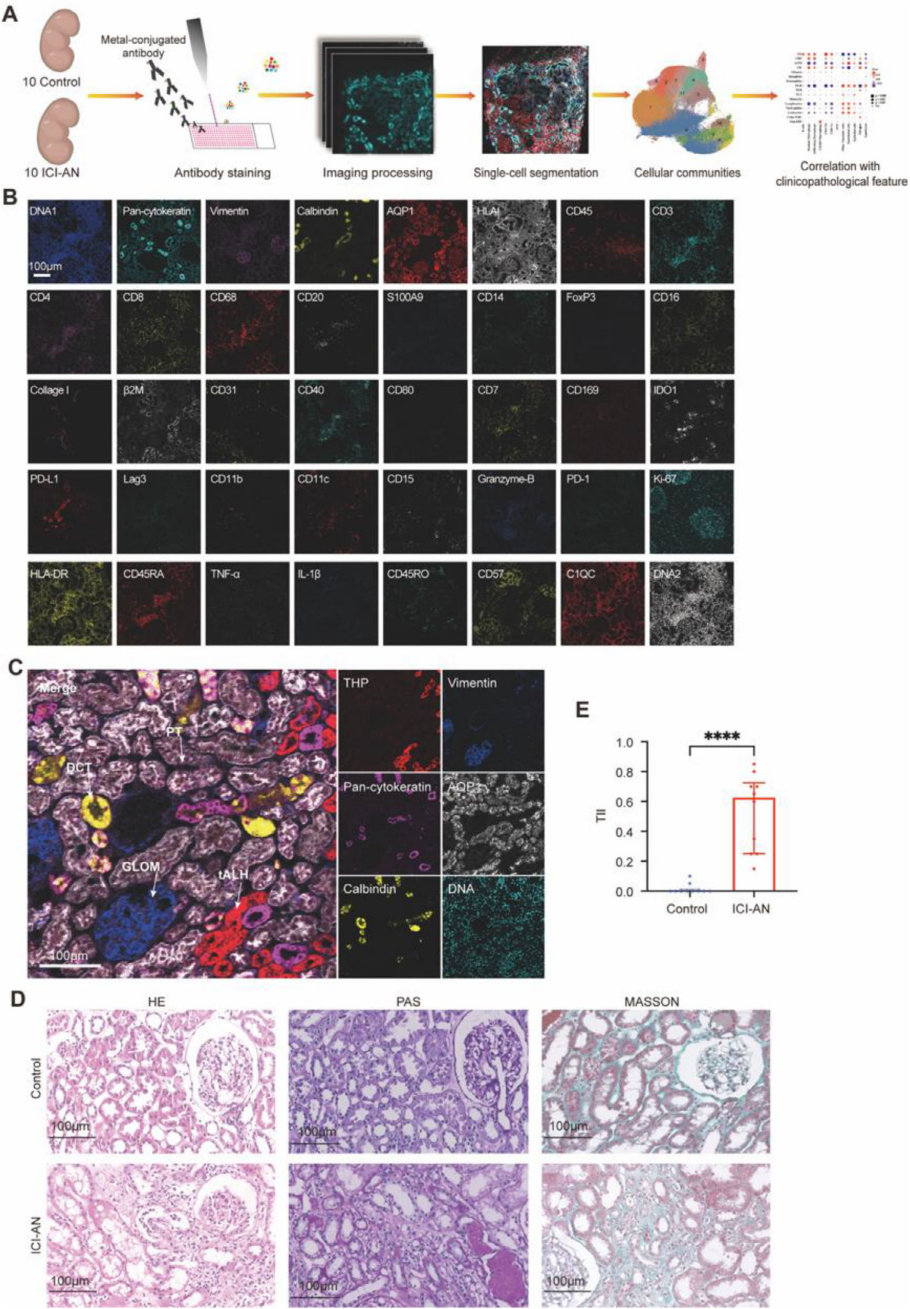
Workflow and imaging analysis of immune checkpoint inhibitor‐associated nephrotoxicity (ICI‐AN) using imaging mass cytometry (IMC). A) Workflow for analyzing tissues from 10 control individuals and 10 patients with ICI‐AN using IMC. The process includes antibody staining, image processing, single‐cell segmentation, clustering, and correlation analysis. B) Representative IMC images of 40 markers. Scale bars: 100 µm. C) Detailed IMC staining for structural and functional markers in kidney tissues. Markers include Aquaporin 1 (AQP1, white) in proximal tubules, Vimentin (blue) in glomeruli, and DNA (green) in nuclei, with Tamm–Horsfall protein (THP, red) marking the thick ascending limb of Henle's loop and Calbindin (yellow) in distal convoluted tubules. Scale bar: 100 µm. D) Representative hematoxylin and eosin (H&E) staining, periodic acid‐Schiff (PAS), and Masson's trichrome staining show increased tubular injury and fibrosis in the ICI‐AN group compared to controls. Scale bars: 100 µm; magnification: 200×. E) Tubular injury index (TII) between ICI‐AN patients and controls (*n* = 10 per group). Data are shown as median with interquartile range. Statistical comparison was performed using the two‐sided Mann–Whitney *U* test due to non‐normal distribution in the control group. ****P < 0.0001.

To investigate ICI‐AN, we employed a comprehensive approach combining IMC, histopathological analysis, and multi‐omics profiling of kidney tissues from 10 healthy individuals and 10 ICI‐AN patients. This workflow included antibody staining, image processing, single‐cell segmentation, and subsequent clustering and correlation analysis (**Figure** **1**A). A panel of 40 antibodies (Table S1, Supporting Information) was used to identify structural and immune markers, enabling spatial identification of renal compartments and immune cell populations. IMC revealed distinct localization of renal markers, such as Aquaporin 1 (AQP1) in proximal tubules, Vimentin in glomeruli, Tamm–Horsfall protein (THP) marking the thick ascending limb of Henle's loop and Calbindin in distal convoluted tubules, along with immune markers (CD45, CD68, CD3, CD8, and CD4) in kidney tissues (Figure 1B,C). This map established by IMC provided an anatomical framework for downstream analyses. The regional localization of immune markers offered critical spatial context for identifying areas of immune cell accumulation and injury. This guided our hypothesis that the tubulointerstitial compartment is a key site of immune‐mediated damage in ICI‐AN, and justified applying spatial proximity and neighborhood analyses in later sections of the study.

Histopathological staining further highlighted tissue remodeling in ICI‐AN, characterized by immune cell infiltration, tubular injury, and interstitial fibrosis, as observed in H&E, PAS, and Masson's trichrome staining (Figure 1D). The TII was significantly higher in ICI‐AN samples compared to controls (*P* < 0.0001) (Figure 1E), supporting the pathological relevance of the IMC‐based observations.

Together, these data provided a spatial and structural foundation that underpinned the mechanistic and functional investigations described in the subsequent sections of this study.

### 2.2 Cellular Composition and Spatial Distribution in Kidney Tissues

**Figure 2:**
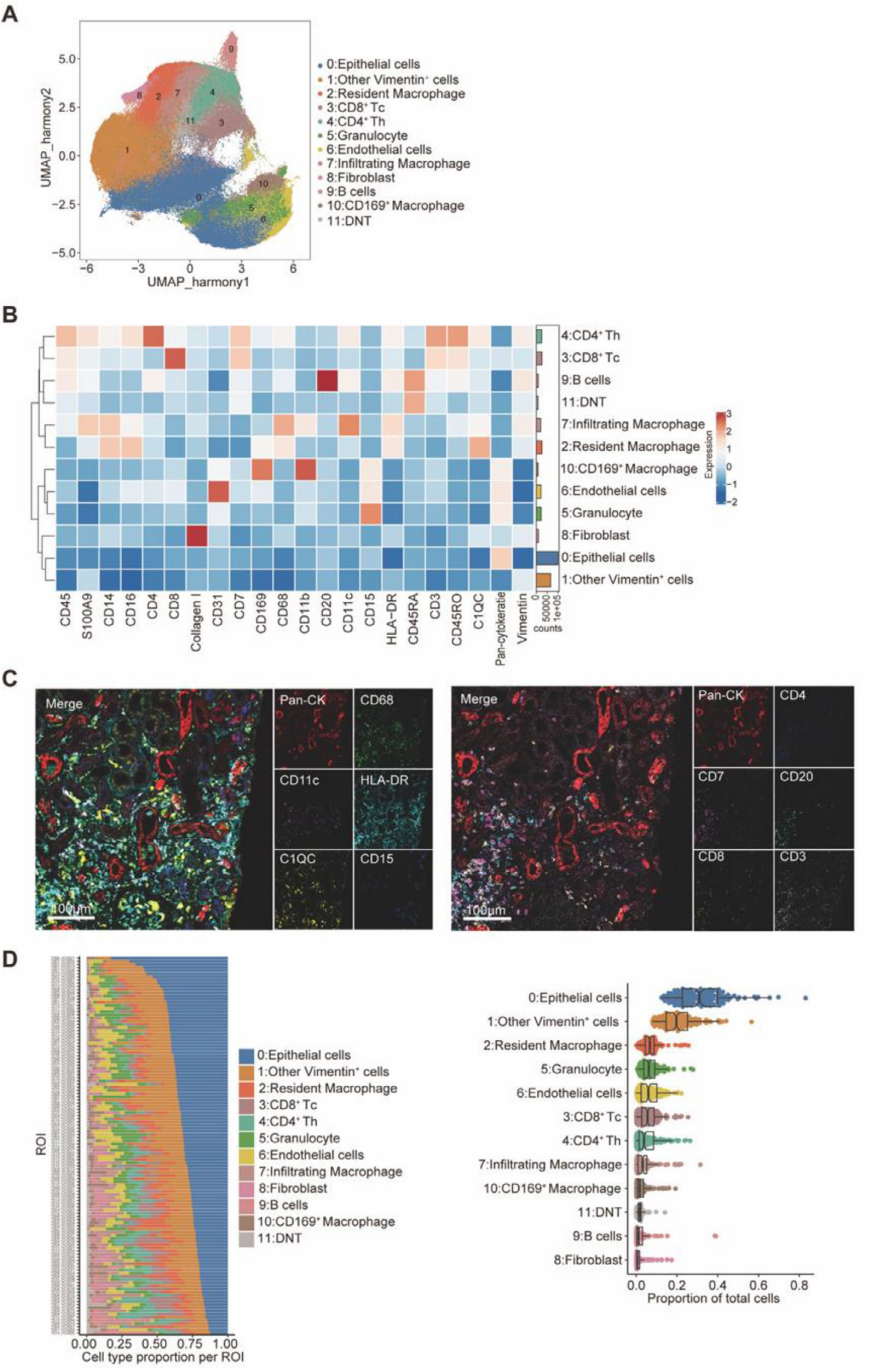
Cellular composition and spatial distribution in kidney tissues. A) Uniform Manifold Approximation and Projection (UMAP) analysis identifying 12 cellular populations within the kidney tissue. B) Expression heatmap of protein markers across identified cell types normalized as *z*‐score. C) Representative imaging mass cytometry (IMC) images showing the spatial distribution of different cell types. Scale bars: 100 µm. D) Proportional distribution of cell populations across regions of interest (ROIs).

Uniform manifold approximation and projection (UMAP) clustering identified 12 distinct cell populations in kidney tissues, including epithelial cells (cluster 0), other vimentin⁺ cells (pericytes, mesangial cells, and podocytes) (cluster 1), resident macrophages (cluster 2), CD8⁺ T cells (cluster 3), CD4⁺ T cells (cluster 4), granulocytes (cluster 5), endothelial cells (cluster 6), infiltrating macrophages (cluster 7), fibroblasts (cluster 8), B cells (cluster 9), CD169⁺ macrophages (cluster 10), and double‐negative T (DNT) cells (cluster 11) (**Figure** **2**A). Heatmap analysis of protein marker expression displayed distinct phenotypic signatures for each cell type (Figure 2B). Spatial mapping showed the spatial organization of immune cells within kidney tissues (Figure 2C), with epithelial cells, other vimentin^+^ cells, and resident macrophages being the dominant populations in both healthy and diseased ROIs (Figure 2D).

### 2.3 Immune Cell Infiltration and Functional Alterations in ICI‐AN

**Figure 3:**
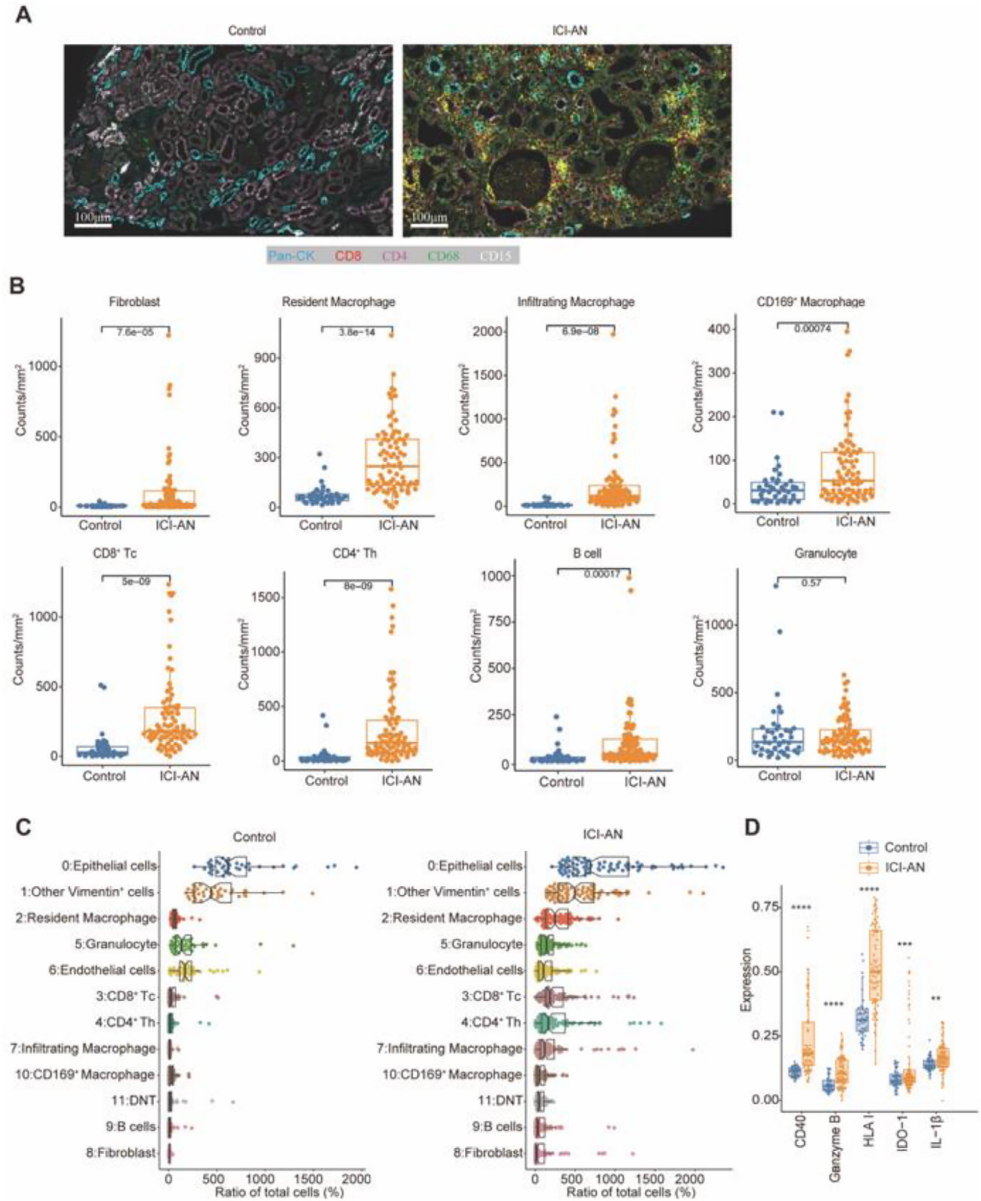
Alterations of immune cell infiltration in immune checkpoint inhibitor‐associated nephrotoxicity (ICI‐AN) patients. A) Representative imaging mass cytometry (IMC) images comparing control and ICI‐AN kidney tissue. Scale bars: 100 µm. B) Quantification and comparison of major cell types between control (*n* = 40 ROIs from 10 individuals) and ICI‐AN (*n* = 80 ROIs from 10 patients). C) Violin plots illustrating the marked increase in resident macrophages in ICI‐AN compared to control kidney tissues. D) Expression profiles of activation and inflammatory markers in resident macrophages from control and ICI‐AN groups. Data are presented as the mean ± SD. Two‐tailed unpaired Student's *t*‐tests. **P < 0.01, ***P < 0.001, ****P < 0.0001.

ICI‐AN kidney tissues exhibited increased immune cell infiltration, predominantly of macrophages and T cells (**Figure** **3**A). To prevent the dilution effect caused by the heavy infiltration of immune cells ICI‐AN, we compared cell density instead of cell frequency. Statistical analysis confirmed significant enrichment of resident macrophages, CD8⁺ T cells, fibroblasts, infiltrating macrophages, CD169⁺ macrophages, CD4⁺ T cells, and B cells in ICI‐AN samples compared to controls (Figure 3B). Resident macrophages represented the most abundant immune population, with a significant increase in ICI‐AN tissues relative to controls (Figure 3C).

Functional characterization of resident macrophages in ICI‐AN tissues displayed an activated phenotype, with elevated levels of CD40 (*P* < 0.0001) and HLA‐I (*P *< 0.0001) (Figure 3D). Activation markers, including Granzyme B (*P* < 0.0001), IDO‐1 (*P* < 0.001), and IL‐1β (*P *< 0.01), were also elevated (Figure 3D). These findings indicated that a resident macrophage‐driven inflammatory microenvironment contributes to tissue injury and fibrosis.

### 2.4 Cellular Interactions and Association with Clinical Features

**Figure 4:**
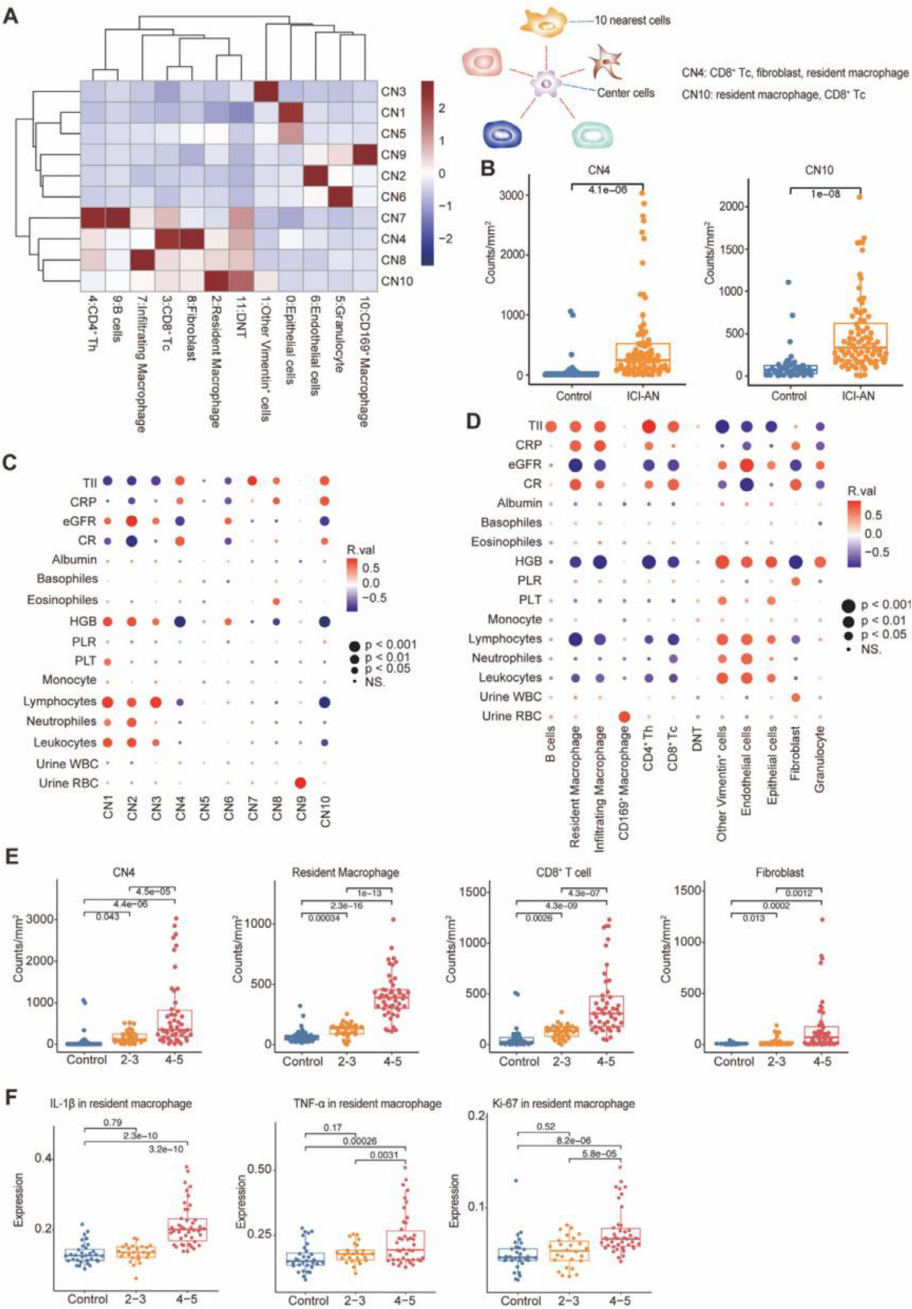
Cellular interactions, and association with clinical features. A) Analysis of cellular neighborhood (CN), highlighting CN4 (CD8⁺ T cell, fibroblast, resident macrophage) and CN10 (resident macrophage, CD8⁺ T cell, fibroblast) for their relevance in immune checkpoint inhibitor‐associated nephrotoxicity (ICI‐AN). B) Quantification comparison of CN4 and CN10 between control (*n* = 40 ROIs) and ICI‐AN patients (*n* = 80 ROIs). C) CN‐to‐clinical parameters interaction analysis in control and ICI‐AN. D) Cell‐to‐clinical parameters interaction analysis in control and ICI‐AN. E) Tubular injury index (TII) severity‐based analysis of cell counts and interactions: control (*n* = 40 ROIs), TII score 2–3 (*n* = 33 ROIs), and TII score 4–5 (*n *= 47 ROIs). F) Marker expression profiles in resident macrophages according to TII score. Data are presented as mean ± SD or as Pearson's correlation coefficients where applicable. Statistical comparisons were performed using unpaired Student's *t*‐test or one‐way ANOVA with Tukey's post hoc test for multiple group comparisons. Correlation analyses were conducted using Pearson's rank correlation.

Cellular neighborhood (CN) analysis revealed distinct immune‐stromal interactions in ICI‐AN tissues (**Figure** **4**A). CN4 (resident macrophage, CD8⁺ T cell, fibroblast) and CN10 (resident macrophage, CD8⁺ T cell) were significantly enriched in nephrotoxic tissues (Figure 4B).

Clinical analysis showed higher serum Cr levels and C‐reactive protein (CRP) levels in ICI‐AN patients than in healthy controls (HCs) (Table S5, Supporting Information). Among the ICI‐AN group, six patients had non‐small cell lung cancer (NSCLC) and four had gastric cancer. All patients received PD‐1 inhibitors, with no cases treated with CTLA‐4 inhibitors alone. Concomitant medication analysis revealed that 3 patients received PPIs, 1 received non‐steroidal anti‐inflammatory drugs (NSAIDs), and 1 received antibiotics during ICI therapy. Histological examination revealed severe inflammatory infiltration, tubular atrophy, and significantly increased interstitial fibrosis in ICI‐AN samples (24.27 ± 5.68%) compared to the control group (5.60 ± 1.4%, *P* < 0.001). CN4 and CN10 abundances were positively correlated with serum Cr, CRP, and TII, while negatively with eGFR (Figure 4C). Specifically, the abundance of resident macrophages and CD8⁺ T cells in CN4 and CN10 showed strong positive correlations with TII, serum Cr, and CRP, and a negative correlation with eGFR (Figure 4D). Similarly, fibroblasts in CN4 were positively associated with serum Cr and CRP, and negatively correlated with eGFR (Figure 4D). Quantitative analysis further demonstrated that CN4 abundance strongly correlated with higher TII scores (Figure 4E). Resident macrophages, CD8⁺ T cells, and fibroblasts were most abundant in areas of severe tubular injury (TII scores 4–5; Figure 4E).

Resident macrophages in ICI‐AN tissues exhibited elevated expression of TNF‐α, IL‐1β, and the proliferation marker Ki‐67, all of which correlated positively with tissue injury severity (Figure 4F). CD8⁺ T cells and fibroblasts also displayed markers of activation, proliferation, and inflammation, as shown in Figure S1 (Supporting Information), further suggesting their contributions to the pathogenesis of ICI‐AN.

### 2.5 ICI‐Induced Nephrotoxicity in Murine Models

**Figure 5:**
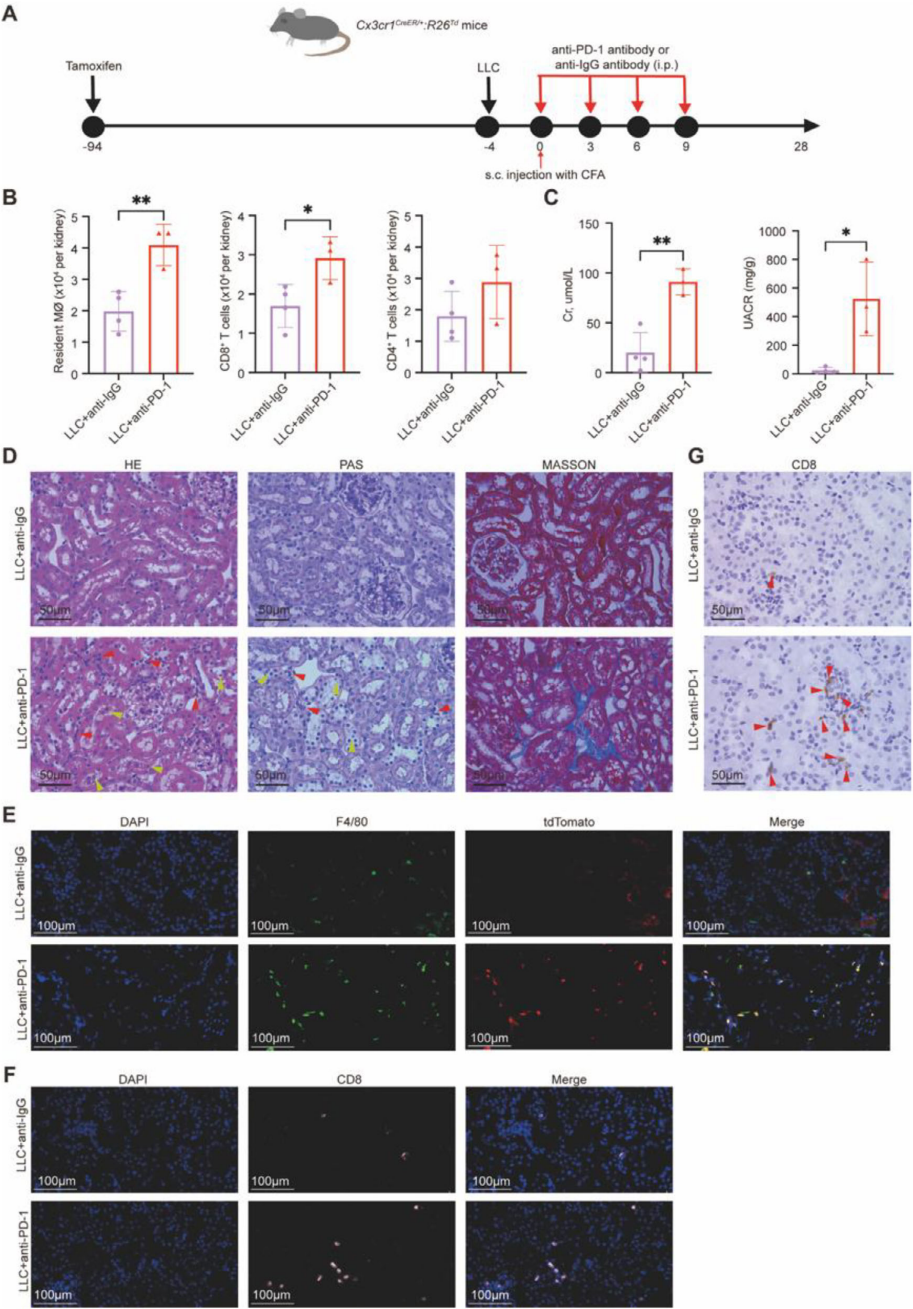
Immune cell infiltration and histopathological analysis of kidneys in mice treated with anti‐PD‐1 antibody. A) Schematic of the experimental timeline in *Cx3cr1^CreER/+^:R26^Td^* mice. CFA: Complete Freund's Adjuvant. B) Quantification of immune cells in the kidney (*n* = 4 and *n* = 3, respectively). C) Serum creatinine (Cr) and urine albumin‐to‐creatine ratio (UACR) levels (*n* = 4 and *n* = 3, respectively). D) Representative histological images of kidney sections stained with hematoxylin and eosin (H&E), Periodic acid‐Schiff (PAS), and Masson's trichrome. Red arrowheads indicate inflammatory cell infiltration, while yellow arrowheads denote areas of tubular injury. Scale bars: 50 µm; magnification: 400×. E) Immunofluorescence analysis showing nuclei (DAPI, blue), macrophages (F4/80, green), and tdTomato⁺ cells (red). Merged images indicate increased resident macrophages in the kidneys of anti‐PD‐1‐treated mice. Scale bars: 100 µm; magnification: 400×. F) Immunofluorescence staining of kidney tissues showing nuclei (DAPI, blue), and anti‐CD8 antibody (pink) to detect CD8⁺ T cells. Scale bars: 100 µm; magnification: 400×. G) Representative immunohistochemistry (IHC) images of CD8⁺ T cells in kidney tissues. Red arrows show CD8⁺ T cell infiltration. Scale bars: 50 µm; magnification: 400×. Data are presented as the mean ± SD. Two‐tailed unpaired Student's *t‐*tests. *P < 0.05, **P < 0.01.

To explore immune mechanisms, we utilized a murine model with tamoxifen‐induced tdTomato labeling of CX3CR1⁺ resident macrophages. Mice implanted with LLC cells were treated with either anti‐PD‐1 or control IgG antibodies (**Figure** **5**A). Anti‐PD‐1‐treated mice displayed increased kidney infiltration of resident macrophages and CD8⁺ T cells compared to controls (Figure 5B; Figure S2A, Supporting Information). Although no notable differences in tumor volume between the groups (Figure S3A, Supporting Information), anti‐PD‐1 treatment significantly elevated serum Cr levels and UACR (Figure 5C). Histological analysis confirmed tubular injury and interstitial fibrosis in anti‐PD‐1‐treated kidneys (Figure 5D), with immunofluorescence and IHC validating increased infiltration of resident macrophages and CD8⁺ T cells (Figure 5E–G).

Notably, the murine model recapitulated key pathological features of human ICI‐AN, including immune cell infiltration, tubular injury, and interstitial fibrosis. In addition, immunofluorescence staining of kidney sections (Figure S2B, Supporting Information) revealed the spatial proximity among CX3CR1⁺ resident macrophages (red), CD8^+^ T cells (pink), and fibroblasts (green), mirroring the immune mechanisms observed in human ICI‐AN.

### 2.6 Resident Macrophages Orchestrate Immune Responses and Fibroblast Activation

**Figure 6:**
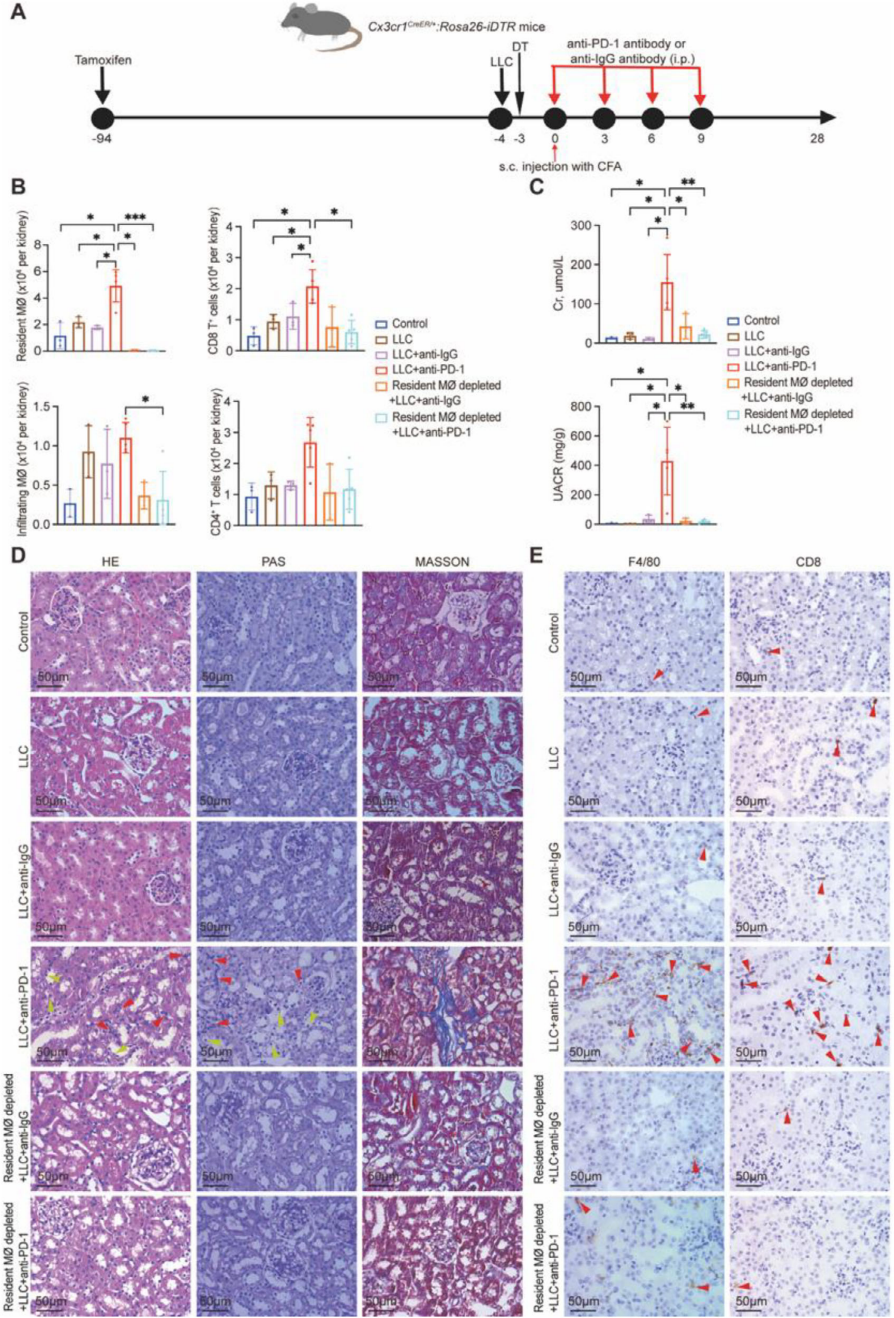
Immune cell infiltration and histopathological changes in response to resident macrophage depletion. A) Experimental protocol in *Cx3cr1^CreER/+^:Rosa26‐iDTR* mice. B) Quantification of immune cells in the kidney. Control, *n* = 3; LLC, *n *= 3; LLC + anti‐IgG, *n* = 3; LLC + anti‐PD‐1, *n* = 5; Resident macrophage depleted + LLC + anti‐IgG, *n* = 3; Resident macrophage depleted + LLC + anti‐PD‐1, *n* = 5. C) Serum creatinine (Cr) and urine albumin‐to‐creatine ratio (UACR) levels across experimental groups. D) Histological images of kidney tissues stained with hematoxylin and eosin (H&E), periodic acid‐Schiff (PAS), and Masson's trichrome staining. Red arrowheads indicate areas of inflammatory cell infiltration, while yellow arrowheads show regions of tubular injury. Scale bars: 50 µm; magnification: 400×. E) Immunohistochemical images of F4/80 (macrophages) and CD8 (CD8⁺ T cells) in different treatment groups. Red arrowheads denote F4/80⁺ macrophages or CD8⁺ T cells infiltration in the kidney tissue. Scale bars: 50 µm; magnification: 400×. Data are presented as mean ± SD. Statistical comparisons were performed using one‐way ANOVA with Tukey's post hoc test. *P < 0.05, **P < 0.01, ***P < 0.001, ****P < 0.0001.

To dissect the role of resident macrophages in ICI‐AN, we utilized resident macrophage‐depletion models (**Figure** **6**A). Tamoxifen‐induced tdTomato labeling allowed specific tracking of CX3CR1⁺ cells, and diphtheria toxin (DT) treatment effectively depleted resident macrophages, as confirmed by flow cytometric gating strategies (Figure S4A,B, Supporting Information). Mice were implanted with LLC tumors and treated with anti‐PD‐1 or control IgG antibodies, with or without resident macrophage depletion, followed by tumor volume assessment and renal immune profiling.

Flow cytometry revealed that anti‐PD‐1 treatment significantly increased resident macrophages and CD8⁺ T cells in the kidneys compared to IgG‐treated mice (Figure 6B). However, in macrophage‐depleted mice, anti‐PD‐1 treatment led to a significant reduction in infiltrating macrophages and CD8⁺ T cell infiltration, demonstrating the critical role of resident macrophages in orchestrating renal immune responses. No significant difference in tumor volume was observed (Figure S3B, Supporting Information). Serum Cr levels and UACR were improved in macrophage‐depleted mice compared to ICI‐treated mice (Figure 6C). Histological analysis showed that depleting resident macrophages significantly reduced inflammatory cell infiltration, tubular injury, and fibrosis (Figure 6D). IHC confirmed increased F4/80⁺ macrophages and CD8⁺ T cells in the kidneys of anti‐PD‐1‐treated mice, which were reduced following resident macrophage depletion (Figure 6E).

### 2.7 Mechanisms Underlying ICI‐AN

**Figure 7:**
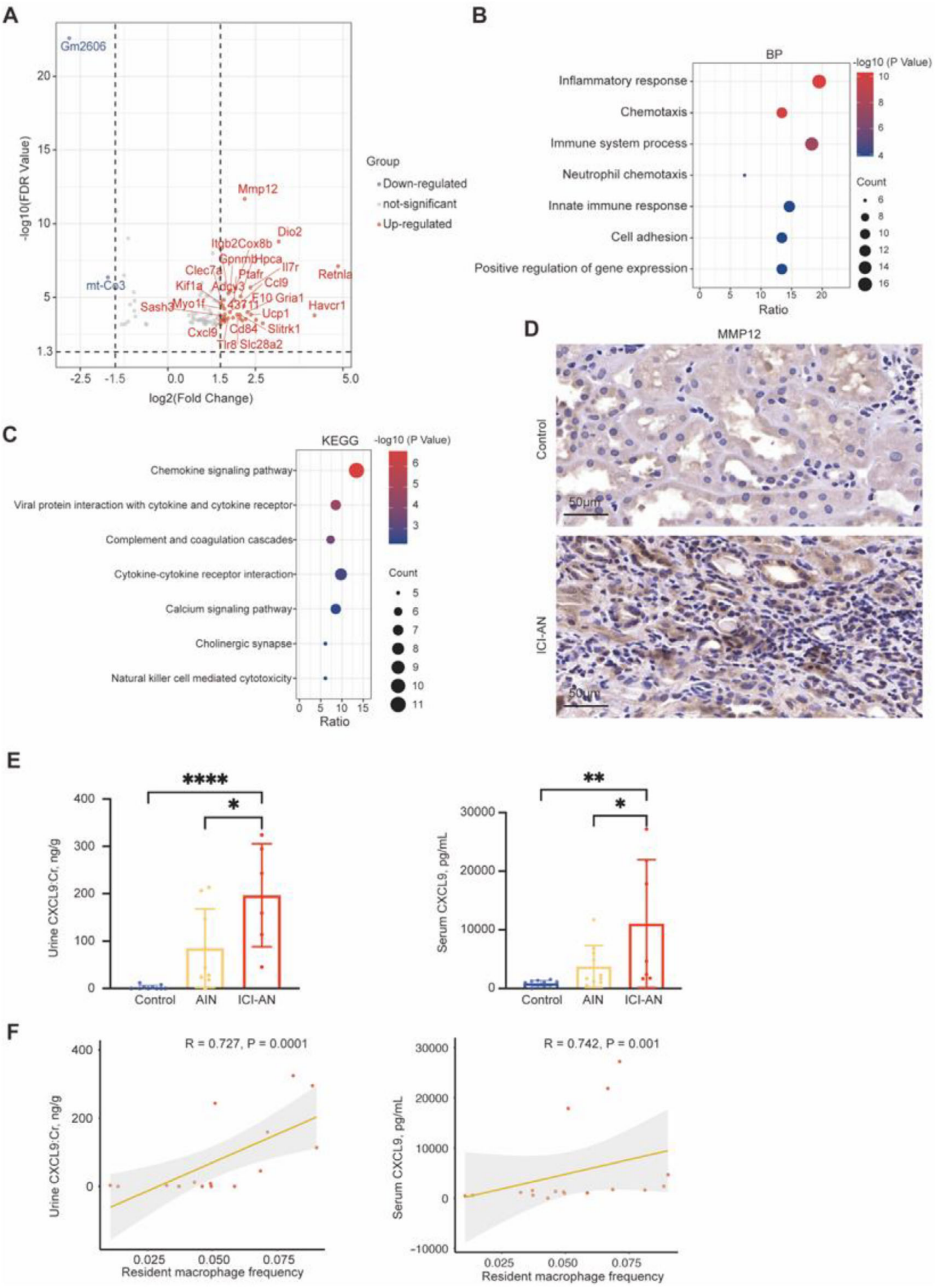
Transcriptomic and protein‐level evidence of MMP12 and CXCL9 upregulation in ICI‐associated nephrotoxicity (ICI‐AN). A) Volcano plot of differentially expressed genes in mouse kidney tissue after ICI treatment, highlighting significantly upregulated genes (e.g., MMP12, CXCL9, CXCL10, and CD74) in red. Differentially expressed genes were identified using a threshold of |log_2_(Fold Change)| > 1.5 and FDR < 0.05. Data were obtained from the GEO database. B) Biological process (BP) enrichment analysis for the upregulated genes in ICI‐AN. C) KEGG Pathway enrichment analysis. D) Immunohistochemical staining of MMP12 in human kidney tissue from control and ICI‐AN group. Scale bars: 50 µm; magnification: 400×. E) CXCL9 levels in urine (*n* = 10 control, *n* = 9 AIN, *n* = 6 ICI‐AN) and serum (*n* = 10 control, *n* = 10 AIN, *n* = 7 ICI‐AN). F) Correlation analysis between urinary or serum CXCL9 levels and resident macrophage frequency. Spearman's rank correlation coefficients (R) and *P*‐values are shown. Data are presented as mean ± SD. Statistical comparisons were performed using one‐way ANOVA with Tukey's post hoc test. *P < 0.05, **P < 0.01, ****P < 0.0001.

Transcriptomic data from mouse kidney tissue were analyzed using datasets from the GEO database (GSE145573). Differential expression analysis identified 98 significantly dysregulated genes, including 83 upregulated and 15 downregulated ones. Key upregulated genes included pro‐inflammatory chemokines and pro‐fibrotic cytokines, such as *Mmp12* and *Cxcl9* (**Figure** **7**A). Gene Ontology analysis revealed enrichment in pathways related to the “inflammatory response”, “chemotaxis”, and “immune system process” (Figure 7B). KEGG analysis further supported this, highlighting dysregulated pathways such as “chemokine signaling”, “cytokine–cytokine receptor interaction,” and “complement and coagulation cascades” (Figure 7C). These results suggested that immune activation and chemokine signaling are key contributors to ICI‐induced renal injury.

IHC of human kidney biopsies confirmed elevated MMP12 in ICI‐AN patients, predominantly in the tubulointerstitial region, coinciding with areas of immune cell infiltration and fibrosis (Figure 7D). Further analysis of CXCL9, a chemokine implicated in macrophage‐driven immune activation, revealed significantly elevated levels in both urine (*P* < 0.0001) and serum (*P* < 0.01) of ICI‐AN patients compared to HCs. Importantly, urinary CXCL9 levels were also markedly elevated in ICI‐AN patients compared to those with acute interstitial nephritis, suggesting that urinary CXCL9 may serve as a specific and sensitive biomarker for ICI‐AN (Figure 7E).

Correlation analysis demonstrated a strong positive association between urinary CXCL9 levels and resident macrophage abundance (*R* = 0.727, *P* = 0.0001), while the correlation with serum CXCL9 was moderate but significant (*R* = 0.742, *P* = 0.001; Figure 7F). Urinary CXCL9 levels showed a strong correlation with serum Cr (*R* = 0.735, *P* = 0.0018; Figure S5A, Supporting Information), while serum CXCL9 also correlated with serum Cr but to a slightly lesser extent (*R* = 0.698, *P* = 0.0024; Figure S5B, Supporting Information). These findings indicated that urinary CXCL9 more accurately reflected local renal immune activation and was a robust biomarker of disease severity.

### 2.8 Resident Macrophages Mediate Kidney Injury via MMP12 and CXCL9

**Figure 8:**
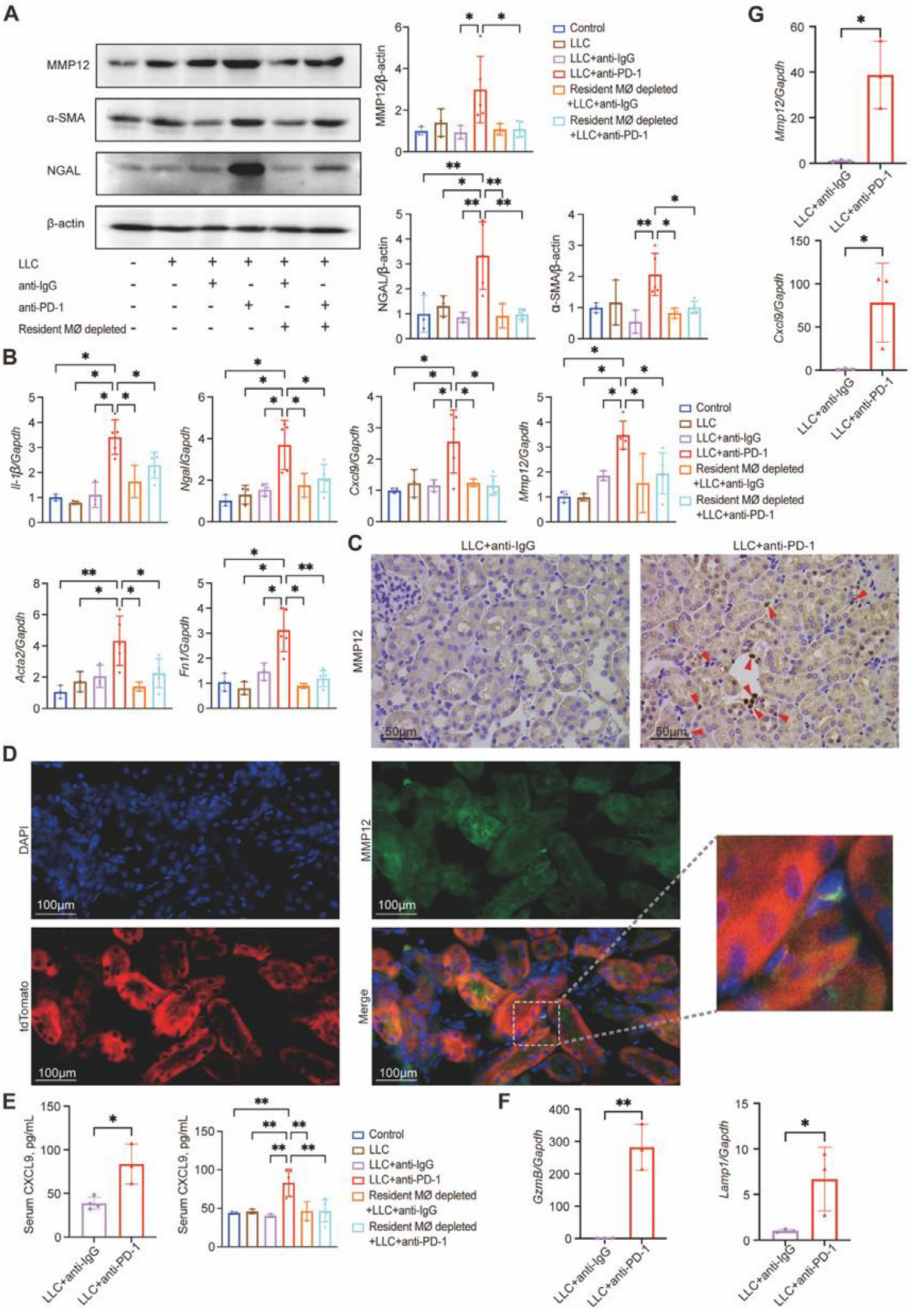
Resident macrophages mediate regional enrichment of MMP12 and induce CXCL9 production. A) Western blot analysis of fibrosis and inflammatory markers. B) Quantitative polymerase chain reaction (qPCR) analysis of pro‐inflammatory and fibrosis‐related genes. C) Immunohistochemical staining of MMP12 in kidney tissue from mice treated with either anti‐IgG or anti‐PD‐1 antibody. Red arrowheads indicate MMP12‐positive cells. Scale bars: 50 µm; magnification: 400×. D) Kidney sections stained with DAPI (blue, nuclei), tdTomato (red, resident macrophages), and MMP12 (green). The merged image shows the colocalization of MMP12 with resident macrophages, highlighted in the magnified region (dashed box). TdTomato signal intensity may vary across figures due to differences in imaging settings and sample preparation. Scale bars: 100 µm; magnification: 630×. E) CXCL9 levels in mouse serum. F) qPCR results of CD8^+^ T cells co‐cultured with resident macrophages (*n* = 3 per group). G) qPCR results of resident macrophages isolated from mouse kidneys treated with anti‐PD‐1 antibody or control IgG (*n *= 3 per group). Control, *n* = 3; LLC, *n* = 3; LLC + anti‐IgG, *n* = 3; LLC + anti‐PD‐1, *n* = 5; Resident macrophage depleted + LLC + anti‐IgG, *n* = 3; Resident macrophage depleted + LLC + anti‐PD‐1, *n* = 5. Data are presented as mean ± SD. Statistical comparisons were made using an unpaired *t*‐test or one‐way ANOVA. *P < 0.05, **P < 0.01, ***P < 0.001, ****P < 0.0001.

Western blot analysis showed that MMP12, α‐SMA, and NGAL were significantly upregulated in the LLC + anti‐PD‐1 group compared to controls, and their expression was reduced upon resident macrophage depletion (**Figure** **8**A). qPCR analysis further confirmed the increased mRNA expression of these markers, including *Mmp12*, *Cxcl9*, *Acta2* (encoding α‐SMA), *Ngal*, *Il‐1β*, and *Fn1*, in LLC + anti‐PD‐1‐treated mice, with reductions observed in the macrophage‐depleted group (Figure 8B). These results suggested that resident macrophages contributed to the inflammatory and fibrotic responses induced by ICI treatment, and were the primary source of MMP12 and CXCL9, as depletion of these cells significantly reduced their expression.

IHC staining further confirmed that MMP12 expression was highly localized to resident macrophages in the interstitial regions of kidneys from anti‐PD1‐treated mice (Figure 8C). Consistent with this, immunofluorescence staining demonstrated that MMP12 (green) colocalized with tdTomato‐labeled resident macrophages (red) in the kidney interstitium (Figure 8D). A magnified view of the merged image clearly demonstrated the localization of MMP12 within tdTomato⁺ macrophages, underscoring their critical role as a primary source of MMP12. Parallel immunofluorescence in LLC + anti‐IgG–treated mice (Figure S6, Supporting Information) revealed minimal MMP12 signal and a lack of colocalization with resident macrophages. Serum CXCL9 levels were similarly elevated in the anti‐PD‐1 treatment group, aligning with observations in human ICI‐AN samples, and were significantly reduced following resident macrophage depletion (Figure 8E).

Co‐culture experiments were performed with sorted resident macrophages from anti‐PD‐1‐treated or control‐treated mice and CD8⁺ T cells from control spleens (Figure S7, Supporting Information). These experiments showed that resident macrophages from ICI‐treated kidneys enhanced CD8⁺ T cells activation, marked by increased *GzmB* and *Lamp1* expression (Figure 8F). Moreover, resident macrophages from ICI‐treated kidneys exhibited higher expression of *Mmp12* and *Cxcl9*, further supporting their role as key drivers of T cell activation and renal immune dysfunction (Figure 8G). Interestingly, infiltrating macrophages did not exhibit significant differences in *Mmp12* or *Cxcl9* expression between groups, suggesting the unique role for resident macrophages in ICI‐AN pathogenesis (Figure S8, Supporting Information).

### 2.9 MMP12 Inhibition and CXCL9 Neutralization Mitigate Renal Injury and Immune Activation in ICI‐AN

**Figure 9:**
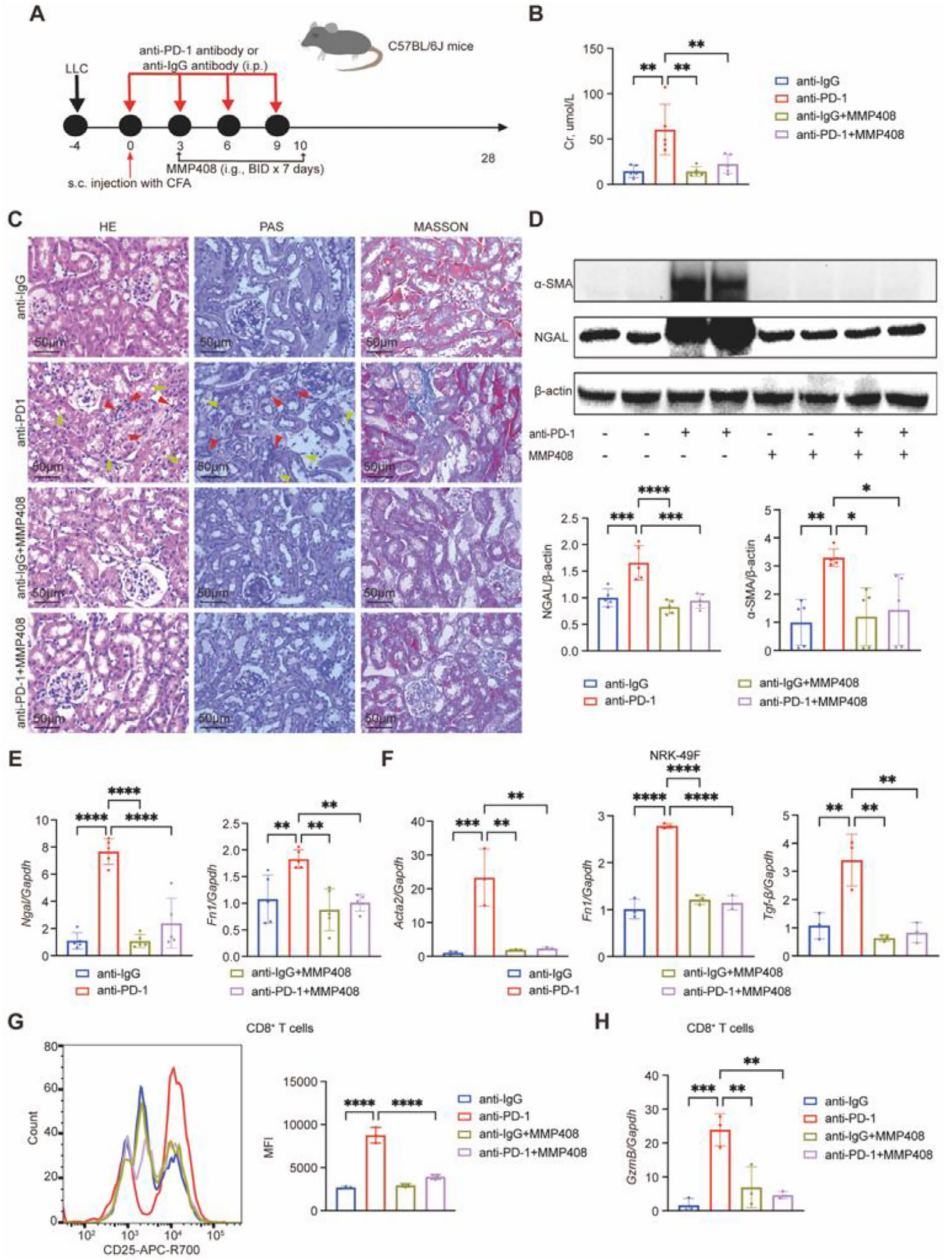
Pharmacological inhibition of MMP12 and neutralization of CXCL9 attenuate renal injury and immune activation in ICI‐associated nephrotoxicity (ICI‐AN). A) Schematic of the experimental timeline in mice treated with MMP408. B) Serum creatinine (Cr) levels in each treatment group (*n* = 5 per group). C) Representative histological images of kidney tissues stained with hematoxylin and eosin (H&E), periodic acid–Schiff (PAS), and Masson's trichrome. Red arrowheads indicate inflammatory cell infiltration; yellow arrowheads indicate tubular injury. Scale bars: 50 µm. D) Western blot analysis of NGAL and α‐SMA protein expression in renal tissues (*n* = 5 per group). E) qPCR analysis of *Ngal* and *Fn1* mRNA expression in kidney tissues (*n* = 5 per group). F) qPCR analysis of fibrosis‐related genes (*Acta2*, *Fn1*, and *Tgfb1*) in NRK‐49F fibroblasts after 48‐hour transwell co‐culture with resident macrophages isolated from anti‐PD‐1‐treated mice, with or without MMP408 pretreatment (*n* = 3 per group). G) Flow cytometric analysis of CD25 mean fluorescence intensity (MFI) on CD8⁺ T cells co‐cultured with resident macrophages in the presence or absence of CXCL9 neutralizing antibody (*n* = 3 per group). H) qPCR analysis of *Gzmb* and *Il1b* expression in CD8⁺ T cells after 48‐hour co‐culture with resident macrophages with or without α‐CXCL9 treatment (*n* = 3 per group). Data are presented as mean ± SD. Statistical comparisons were performed using one‐way ANOVA or unpaired *t*‐test. *P < 0.05, **P < 0.01, ***P < 0.001, ****P < 0.0001.

To explore the therapeutic potential of targeting MMP12 in ICI‐AN, we administered the selective MMP12 inhibitor MMP408 to mice receiving anti‐PD‐1 treatment (**Figure** **9**A). MMP408 treatment led to a significant reduction in Cr levels compared to the anti‐PD‐1 group (Figure 9B), and ameliorated renal histopathology, as shown by H&E, PAS, and Masson's trichrome staining (Figure 9C). Western blot analysis further revealed lower protein levels of NGAL and α‐SMA (Figure 9D). These findings were supported by qPCR, which showed downregulation of *Ngal* and *Fn1* in kidney tissues (Figure 9E).

To assess the downstream impact of MMP12 on macrophage‐fibroblast crosstalk, NRK‐49F fibroblasts were co‐cultured with tissue‐resident macrophages isolated from either control or anti‐PD‐1‐treated mice. Direct addition of MMP408 to the macrophage compartment during co‐culture markedly attenuated the resident macrophage‐induced expression of fibroblast activation genes, including *Acta2*, *Fn1*, and *Tgfb1* (Figure 9F). These results suggested that MMP12 played a central role in promoting fibroblast activation within the ICI‐AN microenvironment.

We next examined whether CXCL9 contributes to resident macrophage‐mediated CD8⁺ T cell activation. CD8⁺ T cells from control mice were co‐cultured with resident macrophages isolated from either control or anti‐PD‐1‐treated mice, in the presence or absence of a neutralizing anti‐CXCL9 antibody. Macrophages from anti‐PD‐1‐treated mice induced strong CD25 upregulation in CD8⁺ T cells, whereas CXCL9 blockade significantly reduced this activation (Figure 9G). qPCR analysis revealed reduced expression of *Gzmb* in CD8⁺ T cells following CXCL9 neutralization (Figure 9H).

## 3 Discussion

This study identified the central role of resident macrophages in mediating immune and fibroblast interactions in ICI‐AN. Using IMC, transcriptomics, and murine models, we elucidated the multifaceted roles of resident macrophages in driving renal inflammation and fibrosis through the production of CXCL9 and MMP12. These findings provided novel mechanistic insights into the pathogenesis of ICI‐AN and open potential avenues for therapeutic interventions to mitigate irAEs.

Our results revealed that resident macrophages were pivotal contributors to ICI‐AN, acting as key mediators of immune activation and tissue remodeling. While previous studies have implicated T cells and macrophages in ICI‐associated renal injury,^[^ ^17^, ^18^, ^19^ ^]^ our research offered a unique spatial and functional characterization of resident macrophages in the kidney. Notably, we identified that PD‐1 blockade triggered the activation of resident macrophages, which secreted CXCL9 to recruit CD8⁺ T cells and produced MMP12 to drive extracellular matrix remodeling and fibrosis. These data suggest that resident macrophages serve as a central nexus linking immune activation and fibroblast‐mediated tissue injury.

CXCL9, an interferon‐γ‐induced chemokine, is widely recognized for its role in T cell recruitment during inflammation responses.^[^ ^20^ ^]^ Previous studies also suggested that CXCL9 may directly contribute to fibrosis.^[^ ^21^ ^]^ Our study demonstrated that elevated urinary CXCL9 levels strongly correlated with resident macrophage abundance and tubular injury severity, indicating its potential as a non‐invasive biomarker for renal immune activation. Importantly, we found that neutralization of CXCL9 in co‐culture experiments significantly attenuated resident macrophage‐induced CD8⁺ T cell activation. However, despite its critical role in immune‐mediated renal injury, CXCL9 blockade has been shown to impair anti‐tumor immune responses in preclinical models,^[^ ^22^ ^]^ suggesting that its clinical utility may lie more in renal‐specific immune monitoring rather than therapeutic inhibition in oncology. In contrast, MMP12, a macrophage‐derived protease, was significantly upregulated in both murine and human ICI‐AN tissues, correlating with fibrosis and tubular injury. Importantly, treatment with a selective MMP12 inhibitor significantly reduced renal injury.

Our findings complement and expand previous research into the mechanisms of ICI‐induced organ toxicity.^[^ ^23^ ^]^ For instance, macrophages have been implicated in ICI‐associated myocarditis and hepatitis, where they mediated immune cell recruitment and tissue damage.^[^ ^24^, ^25^, ^26^ ^]^ However, our study provided a kidney‐specific perspective, highlighting the unique contributions of resident macrophages to ICI‐AN. Unlike other irAEs, such as myocarditis or colitis, ICI‐AN predominantly manifests as acute TIN or immune‐mediated glomerulonephritis. By identifying CXCL9 and MMP12 as central mediators of renal injury, we advanced the understanding of ICI‐AN pathogenesis and offered insights that may not be directly applicable to other organ systems.

One of the most clinically relevant outcomes of this study was the identification of urinary CXCL9 as a potential non‐invasive biomarker for ICI‐AN, consistent with previous findings.^[^ ^27^ ^]^ Urinary CXCL9 levels, reflecting local renal immune activation more accurately than serum levels, correlated strongly with resident macrophage abundance and tubular injury severity. Importantly, urinary CXCL9 levels were significantly higher in ICI‐AN patients than in those with drug‐induced AIN, suggesting its specificity for ICI‐related renal injury. Compared to conventional AKI markers such as serum creatinine, urinary protein, NGAL, KIM‐1, and urinary eosinophils, CXCL9 demonstrated greater specificity for immune‐mediated injury. A summary comparison is provided in Table S6 (Supporting Information). While CXCL9 has previously been associated with drug‐induced AIN, our findings indicate that its further elevation in ICI‐AN may help distinguish it from other forms of AKI. This supports its potential use in diagnosis and monitoring, potentially reducing the need for invasive kidney biopsies. However, its utility requires further validation in prospective, multicenter studies.

Furthermore, our results suggested that MMP12 could represent a promising therapeutic target for ICI‐AN. MMP12, a macrophage‐derived protease, plays a dual role in regulating inflammation and fibrosis.^[^ ^28^, ^29^, ^30^ ^]^ In both in vivo and in vitro models, MMP12 blockade effectively reduced tissue injury and fibroblast activation. Given that MMP12 blockage restrained lung adenocarcinoma metastasis,^[^ ^31^, ^32^ ^]^ targeting MMP12 could potentially provide therapeutic benefits for both renal injury and cancer progression. However, further research is necessary to determine the efficacy and safety of MMP12 inhibition in the context of ICI therapy.

Our findings underscore the critical need to balance effective anti‐tumor immunity with the risk of immune‐related toxicity. While PD‐1 blockade has revolutionized cancer therapy, its off‐target effects, such as ICI‐AN, remain a significant clinical challenge. This study suggested that targeting resident macrophages or their downstream effectors, such as MMP12, could provide a strategy to mitigate renal toxicity without compromising systemic anti‐tumor immunity.

Despite its strengths, this study has several limitations. First, the findings were primarily based on murine models and a relatively small cohort of human samples, which may limit their generalizability. Future studies should validate these findings in larger patient cohorts and explore whether similar mechanisms are involved in other organ‐specific irAEs, such as myocarditis, pneumonitis, and colitis. Second, while our study focuses on resident macrophages, the contributions of other immune cells, such as dendritic cells and B cells, remain to be elucidated. Although the use of a resident macrophage‐specific depletion strategy allowed us to assess their functional role, we acknowledge that this approach may not fully exclude indirect effects mediated by other immune populations. Future studies should explore the broader immune landscape of ICI‐AN to identify additional cellular and molecular players. Third, we employed the LLC model to simulate the NSCLC context, which is relevant since six ICI‐AN patients in our cohort had NSCLC. Although LLC tumors are largely unresponsive to PD‐1 blockade in terms of anti‐tumor efficacy, our primary focus was to model irAEs, particularly kidney injury. Supporting this, a recent study using the same LLC model demonstrated PD‐1 blockade‐induced renal toxicity, highlighting its suitability for studying systemic immune activation and irAEs.^[^ ^33^ ^]^ Nonetheless, given its limited responsiveness to immune checkpoint therapy, future studies using PD‐1–sensitive tumor models—such as MC38 or B16—will be valuable to validate our findings in alternative tumor contexts with more robust anti‐tumor immune responses. Last, the therapeutic potential of targeting CXCL9 and MMP12 requires further investigation in preclinical and clinical settings.

## 4 Conclusion

The study established resident macrophages as central mediators of immune activation and fibrosis in ICI‐AN. By elucidating the roles of CXCL9 and MMP12 in driving renal inflammation and tissue remodeling, we provided novel mechanistic insights into ICI‐AN pathogenesis and identified promising biomarkers and therapeutic targets for this clinically significant irAE. These findings underscore the importance of understanding the immune mechanisms underlying irAEs to develop targeted strategies that enhance the safety of ICI therapy without compromising its anti‐tumor efficacy. Future studies are needed to validate the diagnostic utility of urinary CXCL9 and assess the safety and efficacy of MMP12‐targeted interventions in ICI‐induced toxicities.

## 5 Experimental Section

### Patient Samples and Clinical Data

Kidney biopsy samples were obtained from 10 patients diagnosed with ICI‐AN and 10 age‐ and sex‐matched controls, using healthy donor kidneys collected at the time of transplantation, without a history of renal disease or ICI exposure. Pre‐collected blood and urine samples from these individuals were also analyzed. The research followed the principles of the Declaration of Helsinki and received approval from the Ethics Committee of the First Affiliated Hospital, Zhejiang University School of Medicine (Approval Number: 20241 476). Written informed consent was obtained from all participants.

Clinical data, including serum Cr, estimated glomerular filtration rate (eGFR), and urinary biomarkers, were recorded at the time of biopsy.

### Imaging Mass Cytometry

Formalin‐fixed paraffin‐embedded (FFPE) kidney sections (4‐µm thickness) were used. Samples were deparaffinized with xylene, rehydrated through a graded ethanol series, and subjected to antigen retrieval in Tris‐EDTA buffer (pH 9.2) at 95 °C for 30 min. After cooling, slides were then blocked with 3% BSA in TBS‐T for 45 min, and incubated overnight at 4 °C with a panel of metal‐labeled antibodies (Table S1, Supporting Information). Sections were then washed in TBS and counterstained with Cell‐ID Iridium Intercalator (1:100). After air‐drying, samples were stored at room temperature and analyzed within seven weeks using a Hyperion Imaging Mass Cytometer, with laser ablation performed at 1 µm resolution. Regions of interest (ROIs) covered the entire kidney biopsy section to ensure unbiased tissue sampling. Data were acquired in MCD format and converted to TIFF files for subsequent analysis.

To preprocess IMC images, spillover signal compensation was performed using a previously published spillover matrix.^[^ ^34^ ^]^ Median filtering (3 × 3 kernel) was applied to reduce noise, and contrast was enhanced using the MATLAB function imadjust, redistributing pixel intensities across the 0–255 range. This step improved signal clarity for downstream segmentation.

For image segmentation, a DeepCell‐based algorithm developed by INFI, which identified nuclear (191Ir, 193Ir) and membrane markers (e.g., CD45, CD68) to define individual cells was applied. Additionally, the TissueNet deep learning model^[^ ^35^ ^]^ which uses paired membrane and nuclear channels to achieve human‐level accuracy in single‐cell segmentation is employed. The nuclear signal delineated the nuclei, while membrane markers defined the cellular borders. To validate and compare segmentation results, a connectivity‐aware segmentation pipeline based on MATLAB's regionprops function is also implemented, as described by Wu et al.^[^ ^36^ ^]^ Connected components were identified in each IMC channel, and artifacts were excluded if their nuclear‐to‐membrane centroid distance exceeded 15 pixels. Marker expression levels were used to classify immune, epithelial, and stromal cell populations. Segmentation results were exported as MAT and CSV files for further analysis.

To correct for batch effects, the Harmony algorithm (version 1.2.0) was utilized with parameters optimized based on the dataset distribution. After batch correction, the runUMAP function was applied from the scater package (version 1.28.0) for dimensionality reduction, followed by unsupervised clustering using the fastCluster function from the FastPG package (version 0.0.8). Clusters were manually annotated based on distinct marker expression levels. To compare cell‐type distributions between samples, single‐cell counts were normalized to counts per mm^2^.

For spatial analysis, the 10 nearest neighboring cells were identified for each candidate cell type, based on the methodology described by Sorin M, et al., Nature, 2023.^[^ ^37^ ^]^ This method accounted for cell size variability, avoiding biases introduced by fixed‐radius approaches. The composition of neighboring cells was quantified to define cellular microenvironments. K‐means clustering (*k* = 15) was used to classify cellular neighborhoods, and spatial validation was conducted by overlaying Voronoi allocation diagrams onto the original IMC images.

For marker expression quantification (e.g., IL‐1β, Granzyme B), hyperbolic arc‐sine transformation was applied, and values exceeding the 99th percentile were adjusted to reduce the impact of extreme outliers. Normalization was performed using min–max scaling to obtain relative expression values. To assess spatial interactions, permutation tests were performed to determine whether specific cell types exhibited significant attraction or repulsion. Additionally, patch analysis was conducted to quantify cell distributions across defined distances (0–10, 10–20, and 20–30 µm).

### Histological and Immunofluorescence Analysis

Histological analysis included hematoxylin and eosin (H&E) staining and periodic acid‐Schiff (PAS) to assess inflammation and tubular injury, as well as Masson's trichrome staining to evaluate fibrosis. The tubular injury index (TII) was assessed by two independent pathologists based on histological evaluation of tubular necrosis, loss of brush borders, interstitial inflammation, and fibrosis. Randomly selected high‐power fields (HPFs, ×400) were scored based on the percentage of tubules exhibiting injury, using the following scale: 0 (no injury), 1 (<10%), 2 (10–25%), 3 (26–50%), 4 (51–75%), and 5 (>75%). The average score across at least 10 HPFs was calculated to determine the TII.

Immunofluorescence staining was performed on frozen kidney sections to visualize various markers, including F4/80 (Proteintech, Wuhan, China), CD8 (Abcam, Cambridge, MA, USA), Collagen I (Abcam, Cambridge, MA, USA), and MMP12 (Novus Biologicals, Centennial, CO, USA), as well as their colocalization with tdTomato‐labeled resident macrophages in mice. Antibodies were diluted according to the manufacturer's protocols. Alexa Fluor 488‐conjugated secondary antibodies (Abcam, Cambridge, MA, USA), Opal 650, and Opal 520 secondary antibodies (Akoya Biosciences, Marlborough, MA, USA), were used for the detection. TdTomato fluorescence in resident macrophages was directly visualized. All staining procedures were performed in the dark to preserve fluorescence. Detailed antibody information is provided in Table S2 (Supporting Information). Immunohistochemistry (IHC) was performed on paraffin sections to detect CD8 (Abcam, Cambridge, MA, USA), MMP12 (Novus Biologicals, Centennial, CO, USA), and F4/80 (Cell Signaling Technology, Danvers, MA, USA) expression (see Table S3, Supporting Information).

### Mouse Models

Eight‐week‐old male *Cx3cr1^CreER/+^:R26^T^*, *Cx3cr1^CreER/+^:Rosa26‐iDTR*, or C57BL/6J mice were used in all experiments. *Cx3cr1^CreER/+^:R26^T^* mice were generated by crossing *Cx3cr1^CreERT2^* mice with *Rosa‐stop‐TdTomato* reporter mice. To specifically track resident macrophages, tamoxifen (Sigma‐Aldrich; 75 mg kg^−1^, intraperitoneally) was administered for five consecutive days. Zhu et al. have demonstrated that kidney macrophages consisted of two subsets: bone marrow‐derived infiltrating macrophages, which turn over in ≈46.9 days, and embryo‐derived resident macrophages, which persist long‐term.^[^ ^38^ ^]^ Bone marrow transplantation with torso‐shielded irradiation, as performed in the referenced study, further confirmed that a subset of kidney macrophages was not replaced by circulating monocytes under homeostatic conditions. Given that infiltrating macrophages have a short lifespan (≈2 months), administering tamoxifen 90 days before anti‐PD‐1 treatment ensured that only tissue‐resident macrophages retained tdTomato expression, while transiently infiltrating macrophages were replaced.

*Cx3cr1^CreER/+^:Rosa26‐iDTR* mice were generated by crossing *Cx3cr1^CreERT2^* mice with *Rosa26‐iDTR* mice. Tamoxifen was administered as described above to induce diphtheria toxin receptor (DTR) expression in CX3CR1⁺ cells. To selectively deplete resident macrophages, diphtheria toxin (DT, List Biological Laboratories; 10 ng g^−1^, intraperitoneally) was administered 3 months after tamoxifen treatment, ensuring that only resident macrophages were depleted, while minimizing effects on newly recruited infiltrating macrophages. All transgenic mice were generously provided by Professor Shen's laboratory at Zhejiang University.

For ICI‐AN modeling, mice were subcutaneously implanted with 2 × 10^5^ Lewis lung carcinoma (LLC) cells in the right flank. Mice were treated with 400 µg anti‐PD‐1 antibody or control IgG mixed with Complete Freund's adjuvant (Sigma, F5881) for the first dose, administered subcutaneously at four points (bilateral neck and bilateral inguinal regions). For the additional three doses, anti‐PD‐1 antibody (400 µg) or control IgG was administered intraperitoneally every three days. MMP408 (Sigma‐Aldrich, 444 291) was administered by oral gavage at 5 mg kg^−1^ body weight, twice daily, from day 3 to day 10. The compound was dissolved in a vehicle consisting of 1% DMSO and 2% Tween 80 in sterile 0.9% sodium chloride. Control mice received an equal volume of vehicle solution on the same schedule. All mice were sacrificed on day 28 after the first anti‐PD‐1 administration. The choice of this time point was based on our preliminary time‐course experiments, which demonstrated that renal injury was more severe at 4 weeks than at 3 weeks, as well as on multiple independent studies^[^ ^33^, ^39^, ^40^, ^41^ ^]^ that also used day 28 as the endpoint and reported peak immune‐related tissue damage. Renal function was assessed by measuring serum Cr and UACR. Tumor volumes were measured using calipers, and histological evaluation, flow cytometry, and transcriptomic analysis were utilized to evaluate immune cell infiltration and kidney injury.

All animal procedures were approved by the Institutional Animal Care (Approval Number: ZJCLA‐IACUC‐20010438) and Use Committee and conducted following ethical guidelines for animal experimentation.

### Flow Cytometry

Single‐cell suspensions were obtained from mouse kidneys by enzymatic digestion with DNase I (0.1 mg mL^−1^) and collagenase IV (1 mg mL^−1^) at 37 °C for 30 min, followed by filtration through a 70‐µm cell strainer. Red blood cells were lysed using RBC lysis buffer (eBioscience, San Diego, CA, USA) at room temperature for 3 min, followed by washing with PBS and centrifugation at 300 × g for 5 min to remove debris. For flow cytometry, cells were first stained with live/dead dye (Biolegend Zombie Violet) to exclude dead cells. The viability staining was performed at 4 °C for 30 min in darkness. After staining, cells were washed and incubated with fluorophore‐conjugated antibodies against surface markers CD45, CD3, CD4, CD8, CD11b, F4/80, Ly6G, CD24, MHC II, and Ly6C (eBioscience, San Diego, CA, USA) in FACS buffer (PBS containing 2 mM EDTA and 2% FBS) for 30 min at 4 °C (Table S2, Supporting Information). Absolute cell counting was determined using 123count eBeads Counting Beads (Invitrogen, Thermo Fisher Scientific) according to the manufacturer's instructions. For co‐culture experiments, CD8⁺ T cells were harvested and stained with anti‐CD25 antibody to evaluate activation status. Resident macrophages, CD8^+^ T cells, and infiltrating macrophages were sorted using the MoFlo Astrios sorter for downstream analyses, such as co‐culture experiments and RNA isolation.

### Co‐Culture Experiments

Kidney‐resident macrophages were sorted from anti‐PD‐1‐treated or control‐treated mice. CD8⁺ T cells were isolated from control spleens. Co‐culture experiments were performed using 24‐well transwell plates with a membrane pore size of 0.4 µm (Costar). Resident macrophages were plated in the upper culture chamber, while CD8⁺ T cells or NRK‐49F fibroblasts (CTCC‐001‐0258) were seeded in the lower culture plate. Cells were cultured in RPMI‐1640 medium (Gibco) supplemented with 5% heat‐inactivated fetal bovine serum, 1% penicillin‐streptomycin. After co‐culture, cells were collected for further analysis.

To assess the role of CXCL9 in macrophage‐mediated T cell activation, a neutralizing anti‐CXCL9 antibody (2 µg mL^−1^; R&D Systems, AF‐492‐NA) was added directly to the macrophage compartment at the beginning of the co‐culture and maintained for 48 h. After incubation, CD8⁺ T cells were collected for downstream analysis, including quantitative polymerase chain reaction (qPCR) of *GzmB* expression and flow cytometry‐based assessment of CD25 surface expression.

To evaluate the effect of MMP12 inhibition, MMP408 (10 µg mL^−1^; Sigma‐Aldrich, 444 291) was added to the upper chamber containing macrophages during co‐culture with NRK‐49F cells and maintained for 48 hours. NRK‐49F were then collected for RNA extraction and qPCR analysis of *Acta2*, *Fn1*, and *Tgfb1*.

### Transcriptomic and Molecular Analyses

Total RNA was extracted from kidney tissue using the RNeasy FFPE Kit (Qiagen) following the manufacturer's protocol. RNA quality and concentration were assessed using a NanoDrop spectrophotometer. qPCR was performed to assess the expression of target genes. For mouse‐derived samples, the following genes were analyzed: *Mmp12*, *Cxcl9*, *Acta2*, *Ngal*, *Il‐1β*, *GzmB*, *Lamp1*, and *Fn1*. For rat‐derived NRK‐49F cells, *Acta2*, *Fn1*, and *Tgfb1* were examined. Primer sequences are listed in Table S4 (Supporting Information). cDNA was generated with the PrimeScript RT Reagent Kit (Vazyme), and qPCR was carried out using SYBR Green Master Mix (Vazyme) on a Roche LightCycler 480 system. Gene expression levels were normalized using the 2^−ΔΔCt^ method, with GAPDH serving as an internal control.

Transcriptomic data from mouse kidney tissue were obtained from the Gene Expression Omnibus (GEO) database (GSE145573). This dataset included gene expression profiles of the kidneys of C57BL/6 mice bearing tumors following treatment with a high dosage of ICI or IgG control treatment.^[^ ^42^ ^]^ Differential gene expression analysis was performed using the DESeq2 package in R, with thresholds set at |log_2_(Fold Change)| > 1.5 and false discovery rate (FDR) < 0.05 to identify significantly dysregulated genes.

Protein validation was conducted using Western blotting. Proteins were extracted from kidney tissues using RIPA lysis buffer containing protease inhibitors. Protein concentrations were measured by a BCA Protein Assay Kit (Thermo Fisher). Proteins were resolved by SDS‐PAGE (Bio‐Rad) and transferred to PVDF membranes (Sigma‐Aldrich). Membranes were blocked with 5% non‐fat milk and incubated overnight at 4 °C with primary antibodies targeting MMP12 (Novus Biologicals, Centennial, CO, USA), α‐SMA (Proteintech, Wuhan, China), NGAL (R&D systems, USA), and β‐actin (Proteintech, Wuhan, China), followed by incubation with HRP‐conjugated secondary antibodies. Detailed antibody information is listed in Table S3 (Supporting Information). Signals were detected using enhanced chemiluminescence substrate and imaged on a ChemiDoc system (Bio‐Rad).

### Elisa

Human serum CXCL9 levels were quantified using ELISA kits from RayBiotech, while urinary CXCL9 levels were normalized to urinary creatinine and measured as a ratio (CXCL9/creatinine). Mouse serum and urinary CXCL9, urinary albumin, and urinary creatinine levels were measured using kits from Elabscience. The urinary CXCL9/creatinine ratio, urinary albumin/creatinine ratio were calculated to account for variations in urine concentration.

All assays were performed following the manufacturer's protocols. Absorbance at 450 nm was measured using a microplate reader, and concentrations were determined using standard curves generated from supplied standards.

### Statistical Analysis

Unless otherwise stated, data were presented as mean ± standard deviation (SD). Statistical comparisons between two groups were performed using unpaired Student's *t‐*tests when data met the assumptions of normality and homogeneity of variance, which were assessed using the Shapiro–Wilk and Levene's tests, respectively. For data that violated these assumptions, non‐parametric tests such as the Mann–Whitney U test were used.

For multiple group comparisons, one‐way ANOVA with Tukey's post hoc test was applied when parametric assumptions were satisfied. When these assumptions were not met, non‐parametric Kruskal–Wallis tests were used, followed by pairwise Dunn's tests with Bonferroni correction for multiple comparisons where appropriate. Correlation analyses were performed using Pearson's or Spearman's correlation coefficients, depending on data distribution and relationship type. Pearson was applied when variables were approximately normally distributed and linearly related; Spearman was used when data were non‐normally distributed or showed non‐linear but monotonic trends. Statistical analyses were performed with GraphPad Prism (v10.0). *P*‐value < 0.05 was considered significant. Mouse groups with small sample sizes (e.g., *n* = 3) were restricted to negative or baseline control conditions.

### Ethics Approval Statement

The research was conducted in accordance with the principles of the Declaration of Helsinki and was approved by the Ethics Committee of the First Affiliated Hospital, Zhejiang University School of Medicine (Approval Number: 20241476). All animal procedures were approved by the Institutional Animal Care and Use Committee (Approval Number: ZJCLA‐IACUC‐20010438).

### Patient Consent Statement

Written informed consent was obtained from all participants prior to their inclusion in the study.

## Conflict of Interest

The authors declare no conflict of interest.

## Author Contributions

Y.M., Y.C., and Q.Y. contributed equally to this work. D.C., J.S., and Y.M. conceived and designed the study. Y.M., Y.C., Q.Y., Y.W., N.O., G.W., and Y.Y. conducted the experiments. M.W. was responsible for the pathology evaluations. F.H., D.C., J.S., Y.M., Y.C., Q.Y., and J.C. made contributions to the analysis and interpretation of the data. D.C. and Y.M. carried out the data analysis and drafted the manuscript. D.C. and J.S. had full access to all study data and took responsibility for ensuring the integrity and accuracy of the data and its analysis.

## Supporting information



Supporting Information

## Data Availability

The data that support the findings of this study are available from the corresponding author upon reasonable request.
