# Supplementary material for: Resident Macrophage‐Orchestrated Immune and Fibroblast Interactions in Immune Checkpoint Inhibitor‐Associated Nephrotoxicity
Source: Adv Sci (Weinh). 2025 Aug 14;12(42):e05445. doi: 10.1002/advs.202505445 (PMC12622469; doi:10.1002/advs.202505445)
Supplement: Supplementary file 1 — Supporting Information [file ADVS-12-e05445-s001.docx]

**Supplementary Material**

**Supplementary figures**


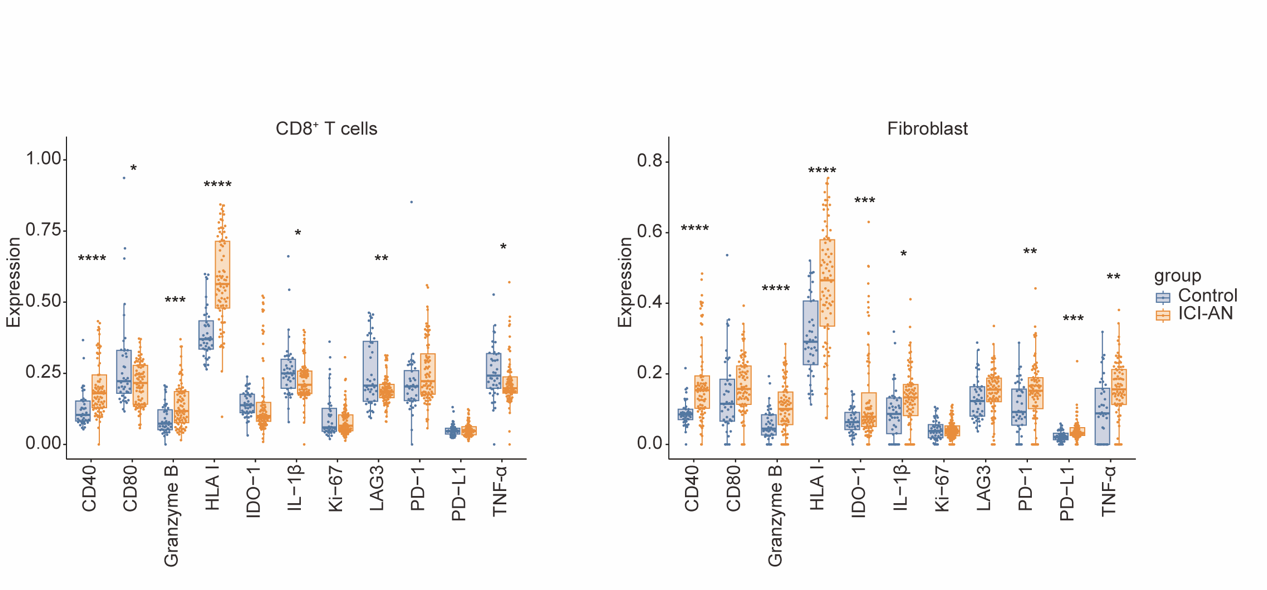


Supplementary figure S1. Analysis of marker expression in CD8⁺ T cells and fibroblasts. Data are presented as the mean ± SD. Statistical comparisons were performed using two‐tailed unpaired Student's t‐tests. *P < 0.05, **P < 0.01, ***P < 0.001, ****P < 0.0001.


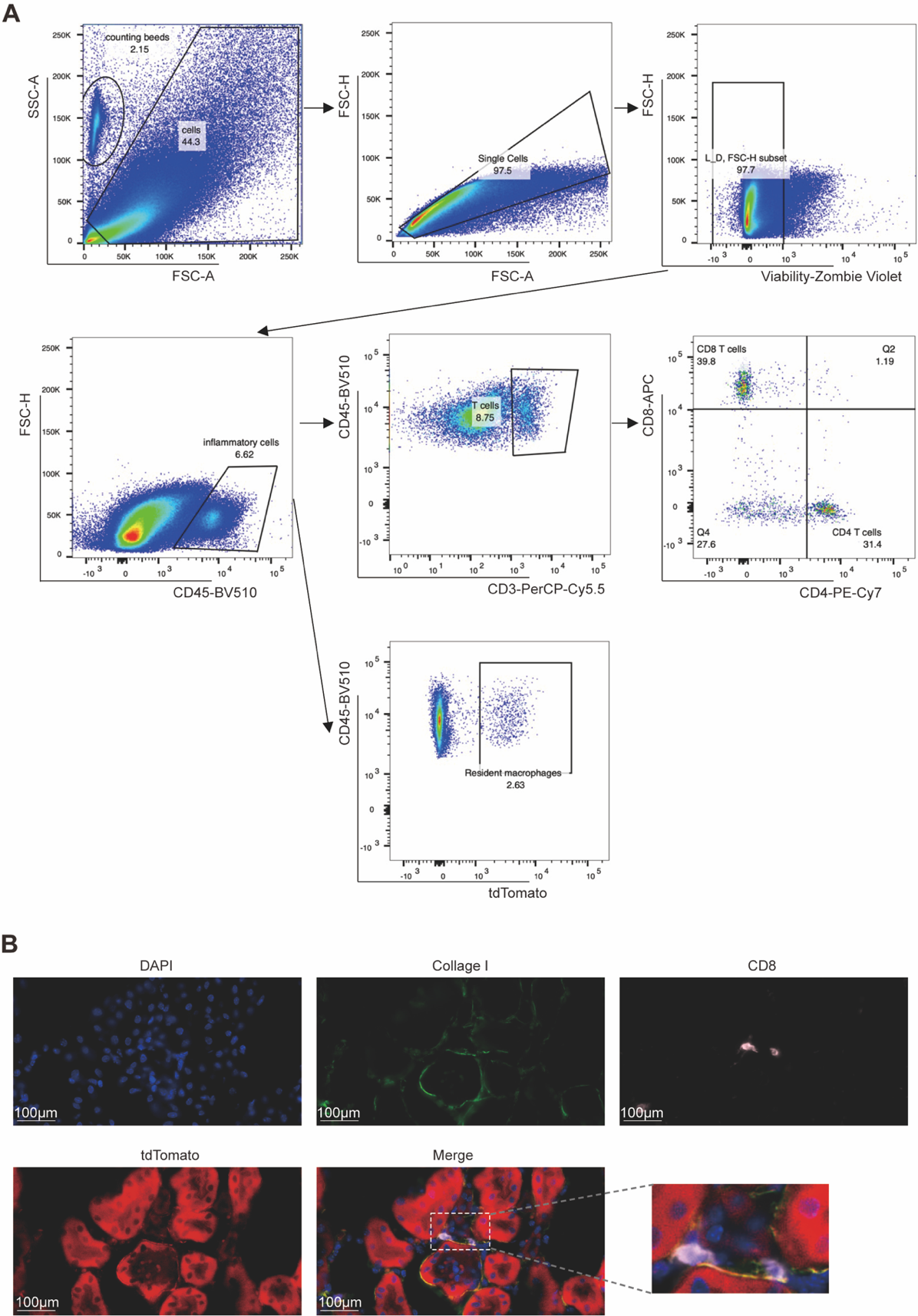


Supplementary figure S2. Identification and spatial localization of immune cells and fibroblasts in kidney tissues.

(A) Gating strategy for flow cytometric analysis of immune cell subsets in *Cx3cr1^CreER/+^:R26Td* mice. (B) Immunofluorescence staining showing DAPI (blue, nuclei), collagen I (green, fibroblasts), CD8 (white, CD8⁺ T cells), and tdTomato (red, resident macrophages). The merged image highlights the close spatial association among these cell types, with a magnified view showing their interactions. Scale bars: 100 μm; magnification: 630×.


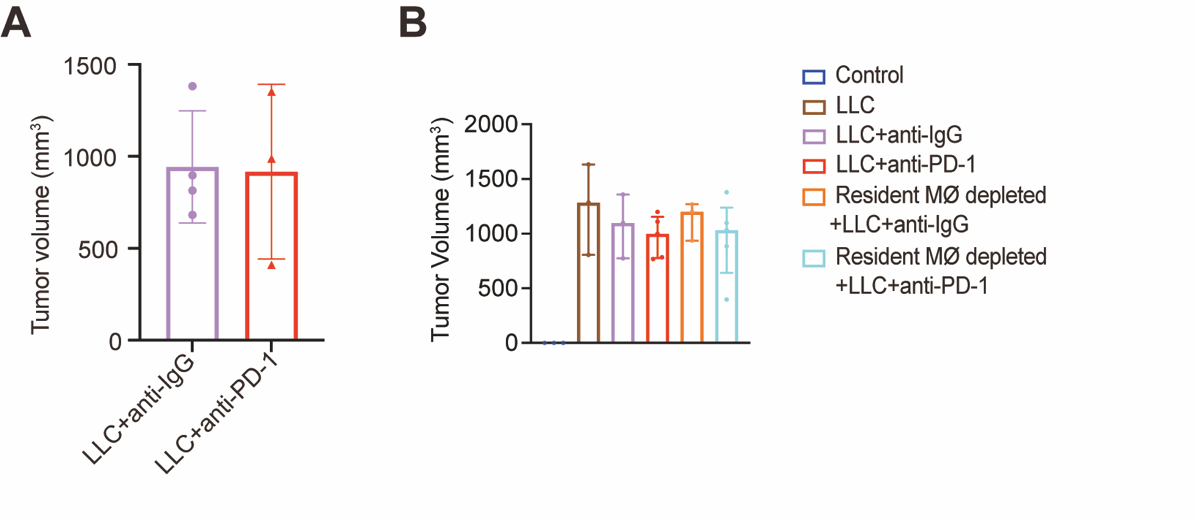


Supplementary figure S3. Comparison of tumor volume in experimental models.

(A) Tumor volume in response to anti-PD-1 treatment in LCC tumor-bearing mice (n = 4 and n = 3, respectively). Data are presented as the mean ± SD. Two‐tailed unpaired Student's t‐tests. (B) Tumor volume following resident macrophage depletion. Control, n = 3; LLC, n = 3; LLC + anti-IgG, n = 3; LLC + anti-PD-1, n = 5; Resident macrophage depleted + LLC + anti-IgG, n = 3; Resident macrophage depleted + LLC + anti-PD-1, n = 5. Data are presented as median with interquartile range. Statistical analysis was performed using Kruskal–Wallis test with Dunn’s post hoc test.


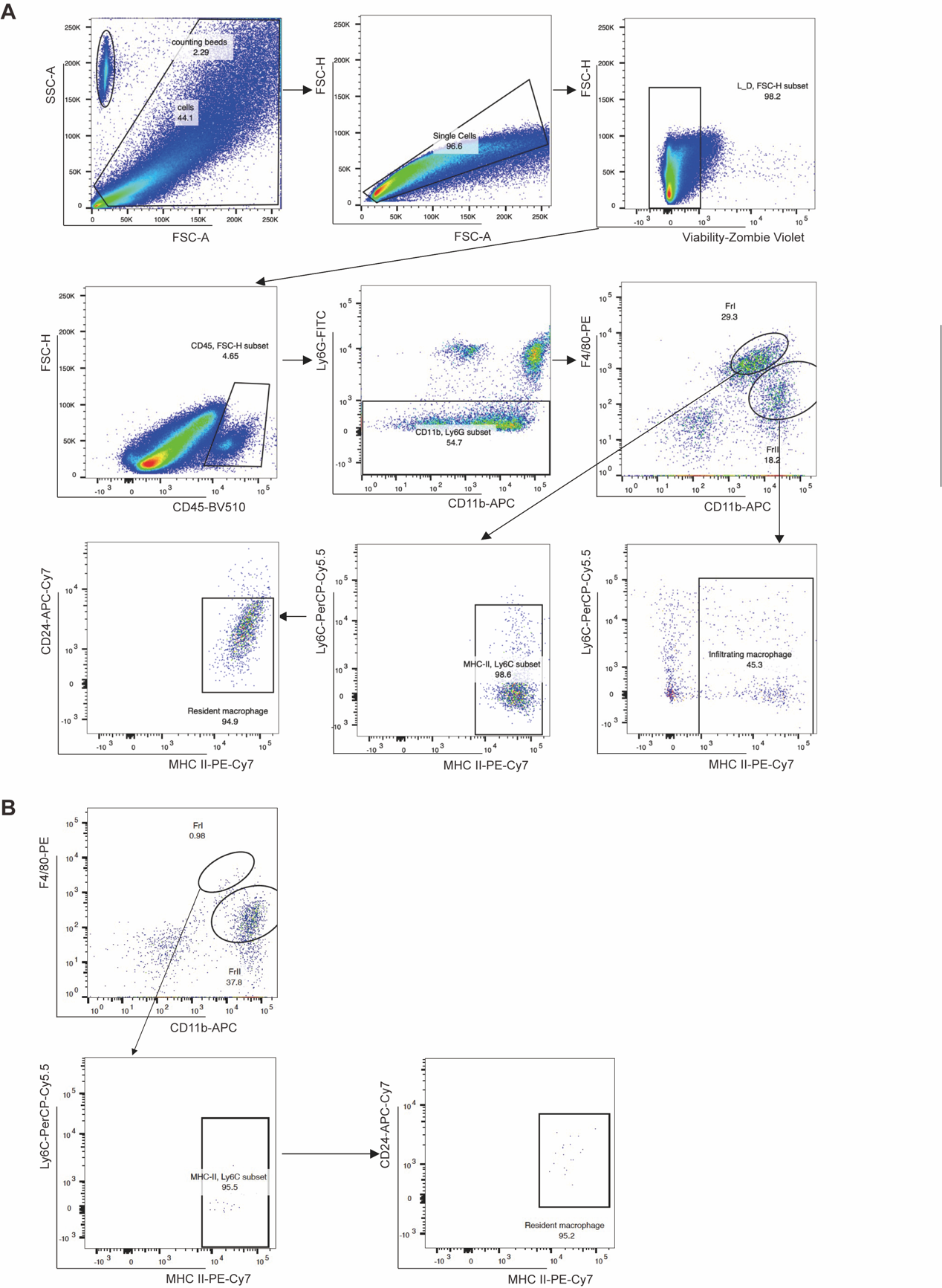


Supplementary figure S4. Flow cytometry gating strategy for characterizing immune cell subsets and verifying the depletion of resident macrophages in *Cx3cr1^CreER/+^:Rosa26-iDTR* mice.

(A) Distribution of resident and infiltrating macrophages by FACS analysis in fresh samples. (B) Validation of resident macrophage depletion using flow cytometry.


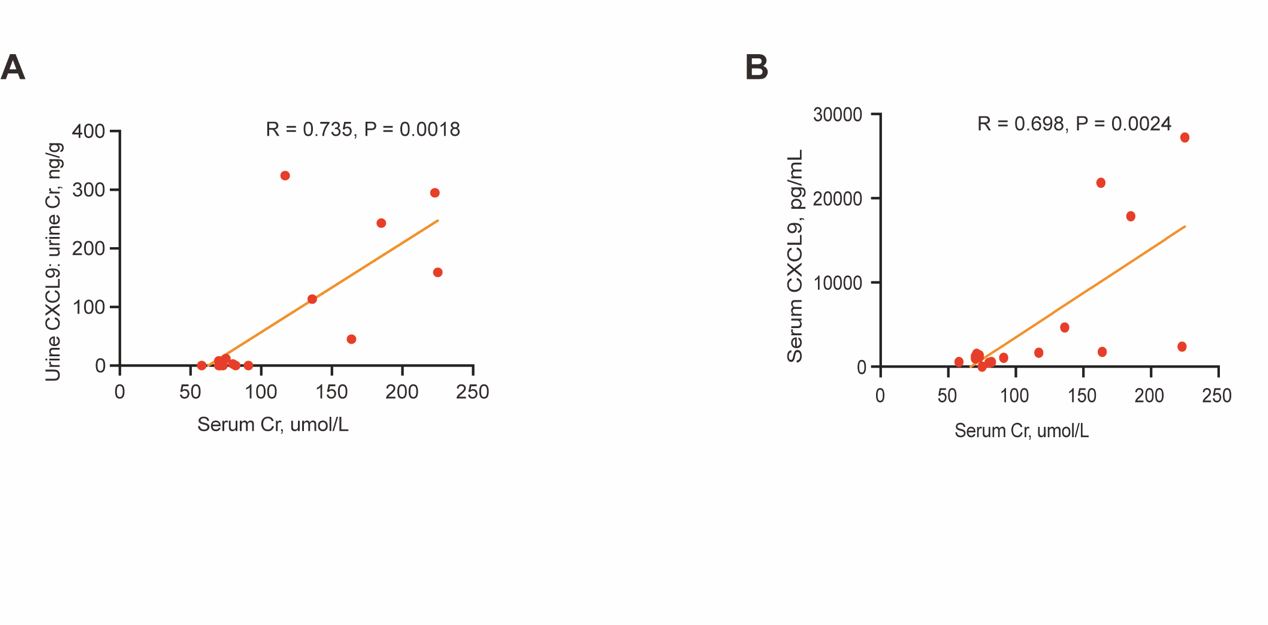


Supplementary figure S5. Correlation between CXCL9 levels and serum creatinine (Cr) levels.

(A) Scatter plot showing the correlation between urine CXCL9 (normalized to urine Cr) and serum Cr levels. A strong positive correlation was observed (R = 0.735, P = 0.0018; Spearman correlation). (B) Scatter plot showing the correlation between serum CXCL9 and serum Cr levels, indicating a moderate but significant positive correlation (R = 0.698, P = 0.0024; Spearman correlation).


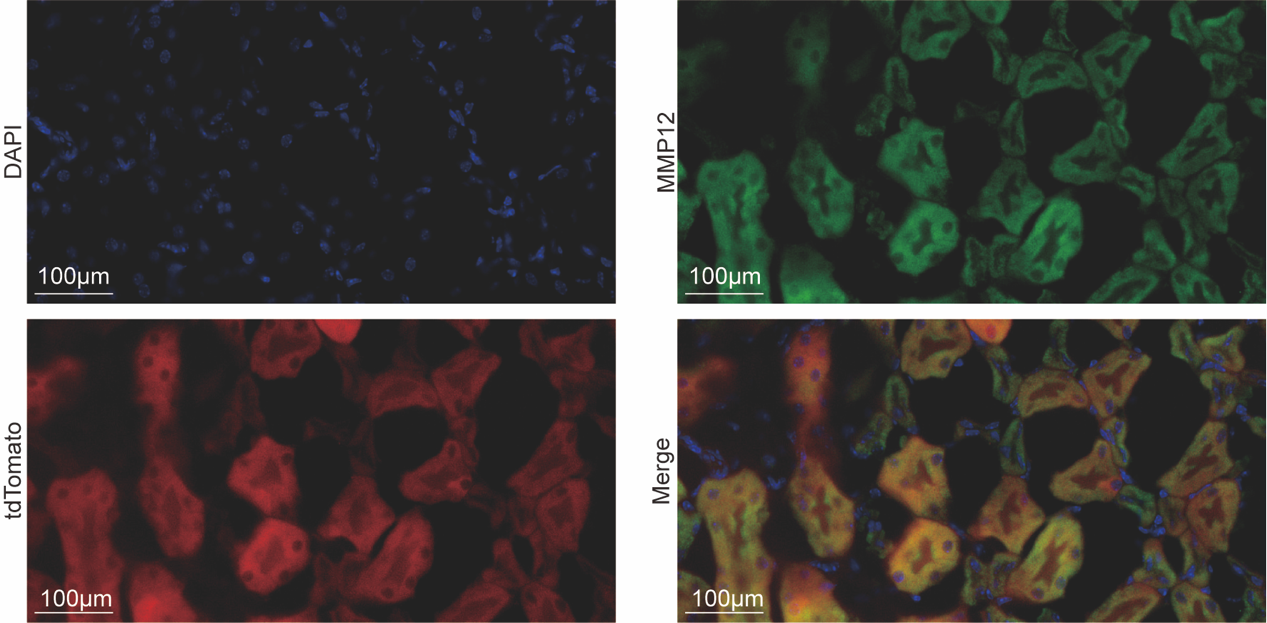


Supplementary figure S6. Baseline expression of MMP12 in kidneys from LLC + anti-IgG-treated mice. Renal tissue stained with DAPI (blue, nuclei), tdTomato (red, resident macrophages), and MMP12 (green). Scale bars: 100 μm; magnification: 630×.


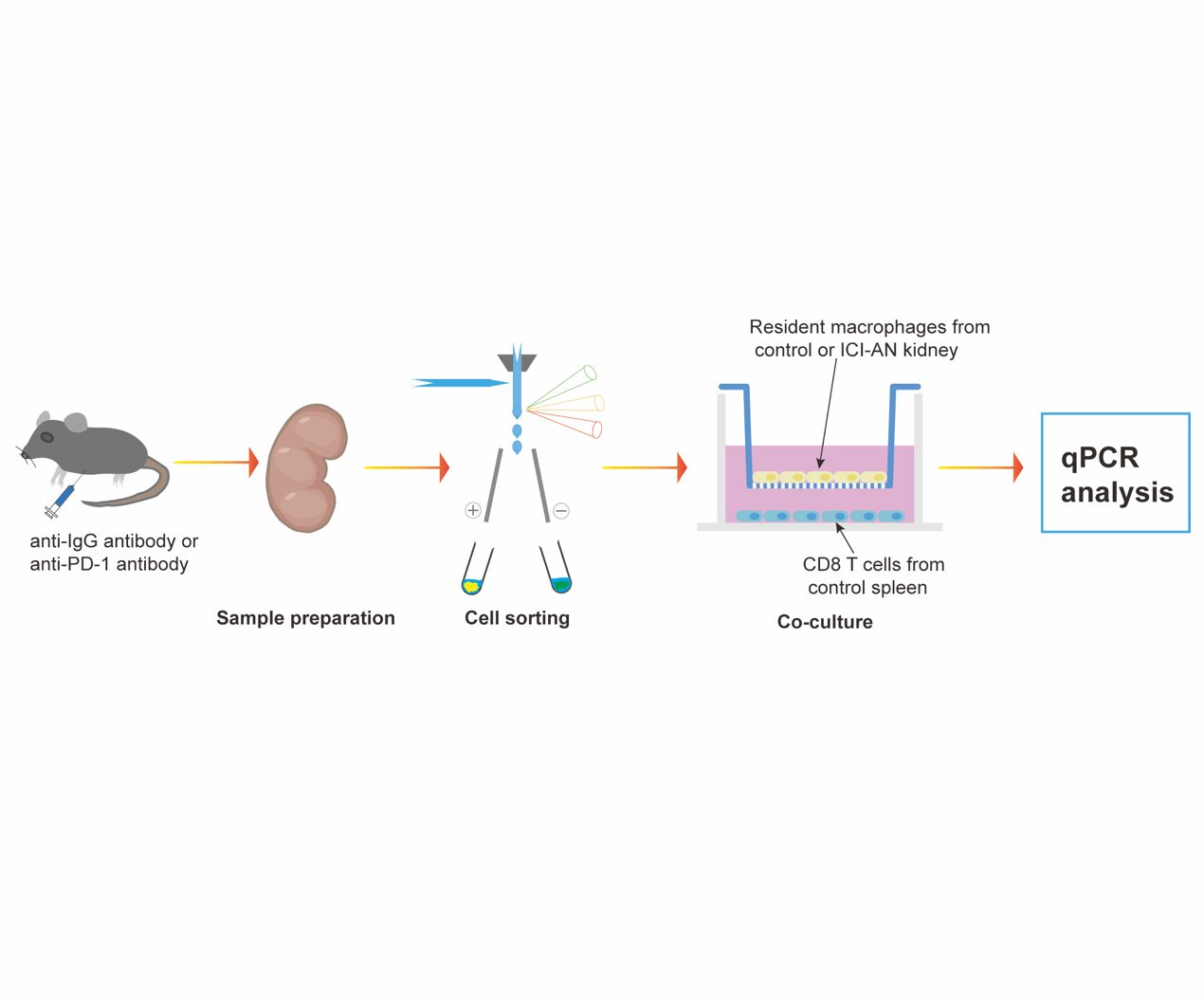


Supplementary figure S7. Workflow for analyzing the interactions between kidney resident macrophages and CD8⁺ T cells in a co-culture system.


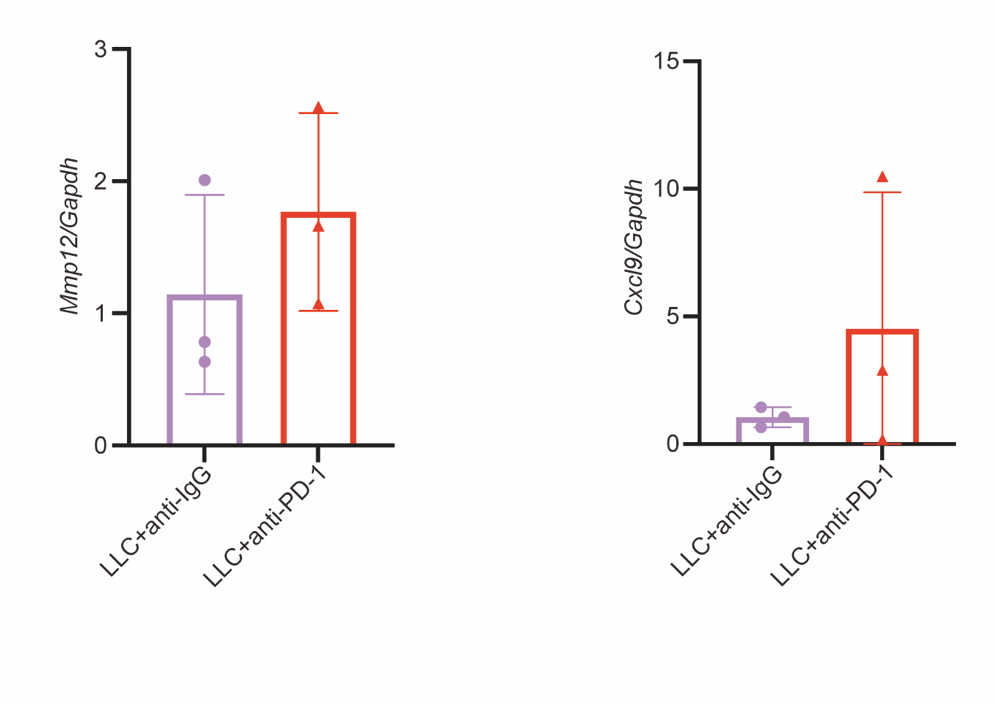


Supplementary figure S8. Expression levels of MMP12 and CXCL9 in infiltrating macrophages isolated from mouse kidneys treated with anti-PD-1 antibody or control IgG (P=0.3661, P=0.3246). Data are presented as the mean ± SD. Statistical analysis was performed using two‐tailed unpaired Student's t‐tests.

Supplementary Table S1: Panel of antibodies for imaging mass cytometry.

| Antibody | Vendor | Catalog number | Clone | Dilution | Metal |
| --- | --- | --- | --- | --- | --- |
| CD45 | CST | 47937SF | D9M8I | 1:800 | 89Y |
| S100A9 | Abcam | ab271864 | EPR3555 | 1:400 | 115In |
| CD14 | Abcam | ab226121 | EPR3653 | 1:400 | 141Pr |
| FOXP3 | CST | 74816SF | D2W8E | 1:100 | 142Nd |
| CD16 | Abcam | ab256582 | EPR16784 | 1:400 | 143Nd |
| HLA I | Abcam | ab239788 | EPR22172 | 1:800 | 144Nd |
| CD4 | Abcam | ab181724 | EPR6855 | 1:200 | 145Nd |
| CD8 | Biolegend | 372902 | C8/144B | 1:400 | 146Nd |
| Collagen I | Abcam | ab215969 | EPR7785 | 1:800 | 147Sm |
| β2M | Thermofisher | 567-MSM5-P1ABX | B2M, 961 | 1:50 | 148Nd |
| CD31 | CST | 85873SF | 89C2 | 1:800 | 149Sm |
| AQP-1 | Abcam | ab178352 | EPR11588(B) | 1:800 | 150Nd |
| CD40 | Abcam | ab271995 | EPR20540 | 1:50 | 151Eu |
| CD80 | Abcam | ab134120 | EPR1157(2) | 1:50 | 152Sm |
| CD7 | Abcam | ab230834 | EPR4242 | 1:50 | 153Eu |
| CD169 | Abcam | ab245735 | SP213 | 1:100 | 154Eu |
| IDO-1 | Proteintech | 66528-1-Ig | 3G2G11 | 1:400 | 155Gd |
| PD-L1 | Abcam | ab226766 | 26938 | 1:50 | 156Gd |
| LAG3 | Abcam | ab241407 | SP346 | 1:50 | 158Gd |
| CD68 | Biolegend | 916104 | KP1 | 1:800 | 159Tb |
| CD11b | Abcam | ab209970 | EPR1344 | 1:800 | 160Gd |
| CD20 | Abcam | ab213033 | IGEL/773 | 1:400 | 161Dy |
| CD11c | Abcam | ab216655 | EP1347Y | 1:400 | 162Dy |
| CD15 | Biolegend | 323035 | W6D3 | 1:800 | 163Dy |
| Granzyme B | Abcam | ab219803 | EPR20129-217 | 1:400 | 164Dy |
| PD-1 | CST | 63815SF | D4W2J | 1:100 | 165Ho |
| Ki-67 | BD | 550609 | B56 | 1:400 | 166Er |
| THP | Novus | MAB5144 | 877914 | 1:400 | 167Er |
| HLA-DR | Novus | NB600-989 | TAL1B5 | 1:400 | 168Er |
| CD45RA | Abcam | ab256137 | 4KB5 | 1:400 | 169Tm |
| CD3 | CST | 24581SF | D7A6E | 1:400 | 170Er |
| TNF-α | Proteintech | 60291-1-Ig | 7B8A11 | 1:200 | 171Yb |
| IL-1β | Proteintech | 66737-1-Ig | 2A1B4 | 1:100 | 172Yb |
| CD45RO | Biolegend | 304239 | UCHL1 | 1:100 | 173Yb |
| CD57 | BD | 555618 | NK-1 | 1:400 | 174Yb |
| C1QC | Abcam | ab247391 | EPR2984Y | 1:400 | 175Lu |
| Pan-cytokeratin | Biolegend | 914204 | AE-1/AE-3 | 1:800 | 176Yb |
| Calbindin | Thermofisher | MA5-24135 | 401025 | 1:400 | 194Pt |
| Vimentin | CST | 46173SF | D21H3 | 1:800 | 198Pt |
| DNAI |  |  |  |  | N/A |

CST: Cell Signaling Technology.

Supplementary Table S2: Antibodies utilized for flow cytometry or immunofluorescence.

| Antibody | Vendor | Catalog number | Clone | Conjugated fluorochrome |
| --- | --- | --- | --- | --- |
| anti-mouse CD45 | BioLegend | 103138 | 30-F11 | BV510 |
| anti-mouse Ly6G | BioLegend | 127605 | 1A8 | FITC |
| anti-mouse F4/80 | BioLegend | 111704 | W20065D | PE |
| anti-mouse/human CD11b | BioLegend | 101212 | M1/70 | APC |
| anti-mouse MHC II | BioLegend | 107630 | M5/114.15.2 | PE-Cy7 |
| anti-mouse Ly6C | BioLegend | 128011 | HK1.4 | PerCP-Cy5.5 |
| anti-mouse CD24 | BioLegend | 101849 | M1/69 | APC-Cy7 |
| anti-mouse CD4 | BioLegend | 100528 | RM4-5 | PE-Cy7 |
| anti-mouse CD8 | BioLegend | 100712 | 53-6.7 | APC |
| anti-mouse CD3 | eBioscience | 551163 | 145-2C11 | PerCP-Cy5.5 |
| anti-mouse CD25 | BD | 565134 | PC61 | APC-R700 |
| F4/80 | Proteintech | 28463-1-AP |  |  |
| MMP12 | Novus | NBP1-31225 |  |  |
| anti-Rabbit IgG | Abcam | ab150077 |  | Alexa Fluor488 |
| CD8 | Abcam | ab217344 |  |  |
| anti-Rabbit IgG | Akoya Biosciences | FP1496001KT |  | Opal 650 |
| Collagen I | Abcam | ab21287 |  |  |
| anti-Rabbit IgG | Akoya Biosciences | FP1487001KT |  | Opal 520 |

Supplementary Table S3: Antibodies for western blot or immunohistochemical studies.

| Antibody | Vendor | Catalog number |
| --- | --- | --- |
| MMP12 | Novus | NBP1-31225 |
| α-SMA | Proteintech | 14395-1-AP |
| NGAL | R&D Systems | AF1757 |
| β-actin | Proteintech | 66009-1-Ig |
| F4/80 | CST | 70076 |
| CD8 | Abcam | ab217344 |

CST: Cell Signaling Technology.

Supplementary Table S4: Primer sequences for quantitative polymerase chain reaction (qPCR).

| Gene symbol | Forward primer | Reverse primer |
| --- | --- | --- |
| Mouse *Gapdh* | AGGTCGGTGTGAACGGATTTG | TGTAGACCATGTAGTTGAGGTCA |
| Mouse *Lamp1* | CAGCACTCTTTGAGGTGAAAAAC | CCATTCGCAGTCTCGTAGGTG |
| Mouse *GzmB* | TCTCGACCCTACATGGCCTTA | TCCTGTTCTTTGATGTTGTGGG |
| Mouse *Mmp12* | GGGCTGCTCCCATGAATGAC | CCAGAGTTGAGTTGTCCAGTTG |
| Mouse *Cxcl9* | GGAGTTCGAGGAACCCTAGTG | GGGATTTGTAGTGGATCGTGC |
| Mouse *Acta2* | CCCAAAGCTAACCGGGAGAAG | GACAGCACCGCCTGGATAG |
| Mouse *Il-1β* | GAAATGCCACCTTTTGACAGTG | TGGATGCTCTCATCAGGACAG |
| Mouse *Ngal* | TGGCCCTGAGTGTCATGTG | CTCTTGTAGCTCATAGATGGTGC |
| Mouse *Fn1* | TTCAAGTGTGATCCCCATGAAG | CAGGTCTACGGCAGTTGTCA |
| Rat *Gapdh* | GCCAAGGTCATCCATGACAAC | GTGGATGCAGGGATGATGTTC |
| Rat *Acta2* | AGCATCCGACCTTGCTAACG | CCAGAGTCCAGCACAATACCAG |
| Rat *Fn1* | GCCCTTACAGTTCCAAGTTCC | AAACCGTGTAAGGGTCAAAGC |
| Rat *Tgfb1* | CGGCAGCTGTACATTGACTT | AGCGCACGATCATGTTGGAC |

Supplementary Table S5: Clinical characteristics of patients with immune checkpoint inhibitor-associated nephrotoxicity (ICI-AN) and healthy controls (HCs).

| Clinical data | HC (n=10) | ICI-AN (n=10) | P value |
| --- | --- | --- | --- |
| Age (years) | 55.70 ± 5.46 | 62.00 ± 7.73 | 0.051 |
| Gender (male/female), n, (%) | 100.00/0 | 100.00/0 | 1.000 |
| Cancer type (NSCLC/gastric cancer), n | NA | 6/4 | NA |
| ICI regimen (PD-1/CTLA-4 inhibitor), n | NA | 10/0 | NA |
| Concomitant medications (PPI/NSAID/  antibiotics), n | NA | 3/1/1 | NA |
| Creatinine at diagnosis (mg/dL) | 73.00 (70.00, 80.50) | 223.50 (158.00, 269.25) | <0.001 |
| CRP (mg/mL) | 1.25 (0.83, 3.51) | 4.37 (3.30, 10.50) | 0.011 |
| Blood eosinophil count (×10^9^/L) | 0.13 (0.10, 0.28) | 0.15 (0.09, 0.21) | 0.912 |
| Haematuria (/HPF) | 3.60 (0.50, 7.40) | 7.35 (4.07, 107.50) | 0.065 |
| Urinary leucocyte count (/HPF) | 2.30 (0.40, 8.20) | 8.70 (3.42, 13.00) | 0.079 |
| Histologic features in kidney biopsy |  |  |  |
| Severe inflammatory infiltrate, n (%) | 0 | 100.0 | <0.001 |
| Severe tubular atrophy, n (%) | 10.0 | 100.0 | <0.001 |
| Interstitial fibrosis (%) | 5.60 ± 1.48 | 24.27 ± 5.68 | <0.001 |
| Presence of eosinophilic infiltrate, n (%) | 0 | 30.0 | 0.211 |
| Presence of plasma cells infiltrate, n (%) | 0 | 20.0 | 0.474 |

NSCLC: non-small cell lung cancer; NA: not applicable; PPI: proton pump inhibitor; NSAID: non-steroidal anti-inflammatory drug; HPF: high power field.

Supplementary Table S6: Comparative overview of CXCL9 and other biomarkers used in the evaluation of ICI-AN.

| Biomarker | Source | Specificity for ICI-AN | Used in clinical practice | Mechanistic relevance | Limitations |
| --- | --- | --- | --- | --- | --- |
| Serum creatinine | Blood | Non-specific | Yes | No | Delayed rise, affected by volume status |
| Urinary protein | Urine | Non-specific | Yes | No | Non-specific marker of injury |
| NGAL | Urine | Non-specific | Limited | No | Elevated in ischemia sepsis |
| KIM-1 | Urine | Non-specific | Limited | No | Elevated in diverse AKI types |
| Urine eosinophils | Urine | Non-specific | Occasionally | No | Poor sensitivity and specificity |
| CXCL9 | Urine/tissue | Elevated in ICI-AN more than AIN | Research only | Produced by macrophages and recruits CD8⁺ T cells | Requires further clinical validation |

ICI-AN: immune checkpoint inhibitor-associated nephrotoxicity.
